# Diamond thin films: a twenty-first century material. Part 2: a new hope

**DOI:** 10.1098/rsta.2023.0382

**Published:** 2025-05-08

**Authors:** Paul W. May, Ramiz Zulkharnay

**Affiliations:** ^1^ School of Chemistry, University of Bristol, Bristol, Bristol, UK

**Keywords:** diamond, chemical vapour deposition, review

## Abstract

Nearly a quarter of a century ago, we wrote a review paper about the very new technology of chemical vapour deposition (CVD) of diamond thin films. We now update this review and bring the story up to date by describing the progress made—or not made—over the intervening years. Back in the 1990s and early 2000s, there was enormous excitement about the plethora of applications that were suddenly possible now that diamonds could be fabricated in the form of thin films. Diamond was hailed as the ultimate semiconductor, and it was believed that the few remaining problems would be quickly solved, leading to a new ‘diamond age’ of electronics. In reality, however, difficulty in making large-area diamond wafers and the elusiveness of a useful *n*-type dopant slowed progress substantially. Unsurprisingly, over the following decade, the enthusiasm and funding for diamonds faded, while competing materials forged ahead. But in the early 2010s, several new game-changing applications for diamonds were discovered, such as electrochemical electrodes, the nitrogen-vacancy (NV) centre defect that promised room-temperature quantum computers, and methods to grow large single-crystal gemstone-quality diamonds. These led to a resurgence in diamond research and a new hope that diamond might *finally* live up to its promise.

This article is part of the theme issue ‘Science into the next millennium: 25 years on’.

## 1. Introduction

In the year 2000, we wrote a review paper entitled ‘Diamond Thin Films: A 21st Century Material’ [1] discussing the relatively new subject of diamond chemical vapour deposition (CVD). The review described what was known at the time about the mechanism of deposition—the limitations of the process—and then speculated about how this technology might develop following a few decades of research. It ended by giving a dozen or so promising applications. Nearly a quarter of a century later, the review has accumulated nearly 1000 citations and has become the ‘go to’ paper for new researchers beginning in this field. So, for this special edition of *Phil. Trans. R. Soc. A*, it is timely to update the original review and evaluate whether the technology has advanced as predicted. Through the use of advanced plasma diagnostics and computer simulation much of the fundamental science underpinning the diamond CVD process—some of which was only speculation at the time—has now been confirmed. However, arguably the most important development in this field over the past 25 years is the astonishing diversity of applications that have now emerged for this material, including medical implants, quantum computing, solar energy generation, optics, electrochemistry, particle detectors, ‘everlasting’ batteries and, of course, the capability to fabricate very large single-crystal diamond (SCD) gemstones that are now challenging natural diamonds for the larger share of the jewellery market [2].

Interest and associated research funding for CVD diamond boomed during the 1990s, fuelled by media hype and unrealistic expectations that the next ‘ultimate semiconductor’ material was imminent. However, the crash soon followed. For a decade from about 2000, the failure to achieve *n*-type doping and frustratingly slow progress in the deposition of large-area diamond wafers dampened enthusiasm for the alleged ‘wonder material’. At the same time, competing materials such as SiC and GaN went from strength to strength, and new exciting wonder materials, such as graphene, promised to outshine even diamond in performance. Funding for diamond research dwindled, until around 2010, when scientists and funding agencies began to realize that the competing materials were also having serious problems of their own—graphene, in particular, having been extremely overhyped [3], meaning diamond was back in the game! This was helped by the discovery that the nitrogen-vacancy (NV) centre in diamond (see §9) could act as a single-photon source, which opened up the almost unbelievable prospect of room-temperature quantum computing. Other exciting applications, such as diamond electrochemical electrodes and biosensors, fuelled diamond’s resurgence, and the spectacular success of synthetic diamond gemstones in the worldwide jewellery market [2] drove the long-awaited technological breakthroughs in diamond growth rate, large-area deposition and defect understanding. Diamond has been recently called the ‘Sleeping Beauty’ of semiconductors [4]—showing outstanding properties with respect to other competing materials but never quite matching their performance in actual commercial devices. It may now be the decade that Sleeping Beauty awakens, bringing with it the new hope that diamond may finally live up to its promise.

Currently, there are over 200 research groups studying the growth or applications of CVD diamonds worldwide and as many as 100 commercial companies fabricating diamonds for gemstone or other applications, or utilizing CVD diamonds in novel applications. As a result, the cost of diamonds has fallen rapidly over the past decade. At the time of writing, the typical cost for a freestanding SCD sample (4 × 4 × 0.5 mm) from a company such as *Element Six* is approximately $2000, while that for a larger (10 × 10 × 0.5 mm) polycrystalline sample is only approximately $50−200 depending on quality, while the number of suppliers has increased accordingly (see electronic supplementary material, table S1).

As one might expect for a ‘follow-on’ paper, to avoid unnecessary duplication of content, it is recommended that the reader first become familiar with the original paper [1] before starting this one. It is also worth pointing out that due to page limitations, only a few of the most important CVD diamond applications are discussed below. However, another recent review covers the vast range of other applications for this material in some detail [5].

## 2. CVD

To recap very briefly, in the CVD process [1,6], a diamond coating is deposited on to the surface of a suitable substrate, for example, a smaller diamond ‘seed’ in the case of gemstone growth, a Si wafer for electronic devices, a quartz window for optics, or a mechanical component for wear applications. This substrate is positioned on a heated stage inside a vacuum chamber and heated to 700–1000°C while process gases are passed over the substrate at a pressure of 20−300 torr. The gas mixture typically consists of a carbon source, such as methane, diluted to a few per cent input mole fraction in hydrogen, although small amounts of other gases such as O_2_, N_2_ or dopants such as B_2_H_6_ or PH_3_ are sometimes added depending on the required product. This gas mixture is energised using either a heated metal (W, Ta or Re) filament placed a few millimetres above the substrate surface or by application of a microwave (MW) discharge in the form of a ‘plasma ball’ that sits above the substrate surface. The thermal or electrical energy deposited into the process gases fragments the molecules to form a chemical ‘soup’ of atoms, radicals, ions and clusters near the substrate surface. Reactive species (mainly H atoms and CH_3_ radicals) from this hot gas mixture diffuse to the surface, and if the conditions are correct, they adsorb on to the surface, migrate around and eventually form a continuous layer of diamond. The diamond coating can remain on the surface after growth and be utilized *in situ*, or the substrate can be chemically etched away to leave a freestanding diamond foil or ‘wafer’, or in the case of thick single crystals, undergo further processing (laser cutting and polishing) to produce synthetic diamond gemstones for the jewellery market.

## 3. Methods for production of diamond CVD

In 2000, when the original review was written, the technology required for diamond CVD was only about 10 years old, and four main deposition techniques were in use: hot filament CVD (HFCVD), MW plasma-enhanced CVD (MWCVD), arc-jets (plasma torches) and oxyacetylene welding torches. In the intervening years, both torch methods have largely fallen out of use in the West. Although extremely high diamond growth rates (approx. 1000 μm h^−1^) could be achieved with these systems [7], problems with substrate cooling, poor uniformity over areas greater than approximately 5 × 5 mm^2^ and inadequate run-to-run reproducibility meant that these methods fell out of favour compared to the more reliable plasma systems. Nevertheless, the easily accessible, bright, visible emission from these torch systems permitted important fundamental studies to be performed on the gas chemistry and growth mechanisms, using techniques such as optical emission spectroscopy (OES) and cavity ring-down spectroscopy [8–10]. These studies provided early empirical data that would later be essential in developing models of the CVD process (see §4).

In China, it was a different story. Arc-jet plasma deposition systems continued to be developed, and this technology is now considered fully mature and commercialized [11]. A series of direct current (DC) arc jets with powers up to 100 kW [12] were developed for different applications, which are capable of mass-producing polycrystalline diamond wafers of diameters up to 150 mm and thickness of approximately 3 mm. Using this technique, the Chinese company, Hebei Plasma Diamond Technology Co. Ltd, has become the main supplier of freestanding diamond film products in the world.

### (a) HFCVD

This technique remains popular among university research groups, mainly for its low cost, versatility, and especially its ability to deposit diamonds over large areas—something with which most other methods still have difficulty. It is now possible to buy commercial HFCVD reactors that deposit polycrystalline diamond uniformly on to Si wafers of area up to 0.5 m^2^ [13], although the low deposition rates (approx. 1 μm h^−1^) mean that thick (greather than 100 μm) large-area diamond films remain rather expensive to manufacture and produce. Smaller HFCVD reactors for sample areas of approximately 100 mm^2^ can be built in-house from standard vacuum components for as little as $20,000, and nowadays many small research groups have their own bespoke diamond HFCVD reactor to study niche research areas. In China, over the past 10 years, HFCVD reactors have been scaled up and modified by biasing the substrate in a technique known as electron-assisted HFCVD [14], together with adopting closely packed filament multi-arrays and short filament-to-substrate distances. As such, HFCVD has become the most important and dominant technique in China for the mass production of thick freestanding polycrystalline diamond films for mechanical and thermal applications [11].

### (b) DC plasmas

These systems were first reported for diamond CVD in 2012 [15], but they remain the ‘poor relation’ among plasma deposition systems, with only a few research groups adopting this as their growth method of choice, mainly at Jilin University in China [16] and in South Korea [17]. DC plasma systems do not require the growth chamber to be a tuned cavity, which is a major drawback with the design of MW plasma systems (see §3d). This greatly simplifies the design of DC reactors and affords the advantage of easier scale-up to larger-area samples. However, the high-power supplies required are expensive, and the growth conditions are not as flexible as those in MWCVD systems, hence their continued relative unpopularity.

### (c) Distributed antenna systems

Rather than use one large plasma source, a number of alternative reactor designs have been developed in the past decade that generate several smaller plasmas distributed in an array or grid over a large area. These distributed plasma reactors have two major advantages over other CVD methods. First, it makes possible very large deposition areas, greater than 1 m^2^. Second, the deposition temperatures (400°C) are typically much lower than those in conventional HFCVD or MWCVD systems, allowing diamond deposition on to substrate materials with low melting points. Indeed, addition of O-containing gases into the feed-gas mixture has been reported to further reduce the growth temperature to 130°C [18]. There are downsides however, the lower deposition temperatures mean that the diamond quality is poor, being mostly nanocrystalline, and growth rates are extremely low (a few 10s of nm h^−1^). Nevertheless, such systems have enabled thin ( less than 100 nm) nanodiamond films to be deposited on to large sheets of glass or plastic for use as protective layers—which would be impossible by other methods.

#### (i) Linear antenna plasma deposition

**Figure 1.**
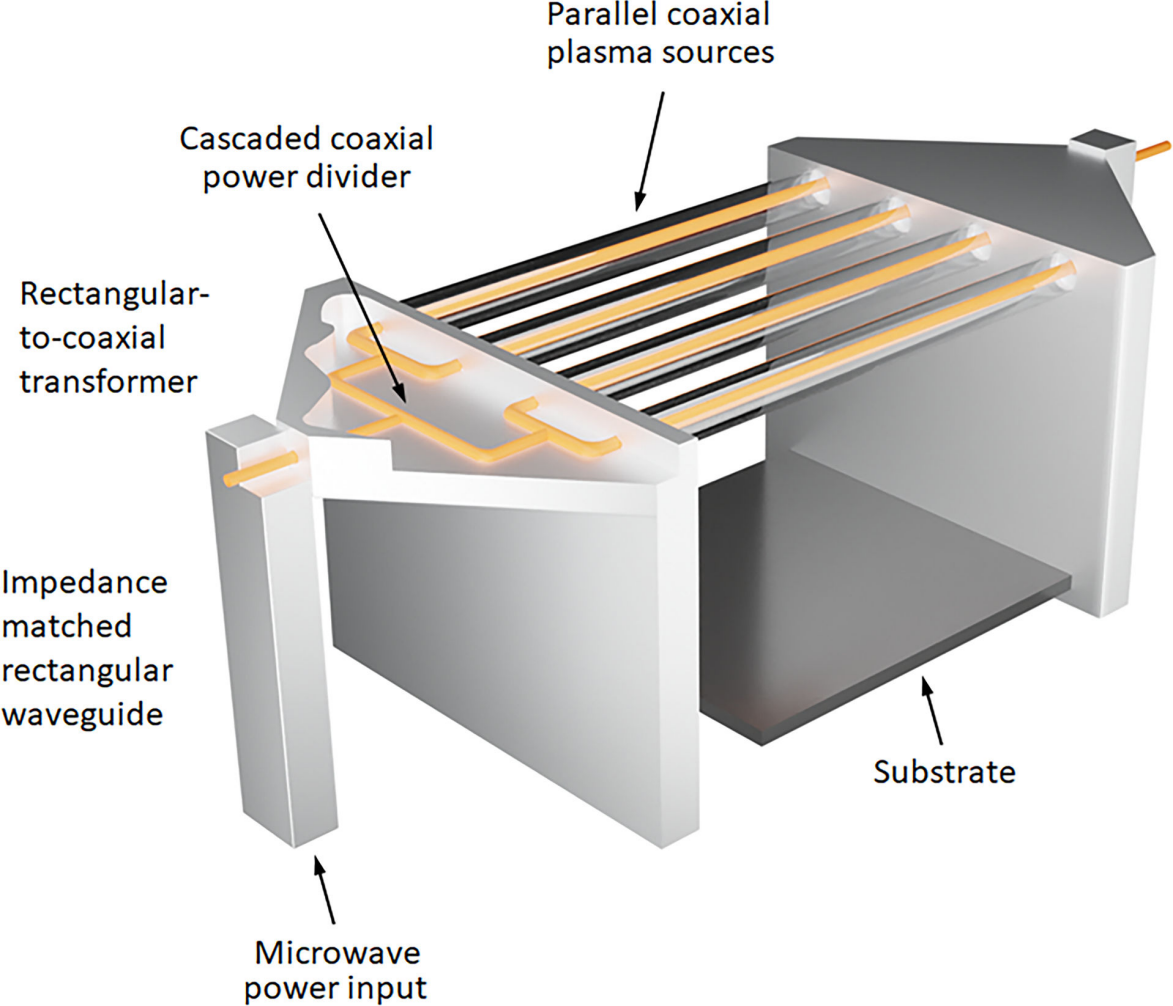
Diagram of a LAPD system. Redrawn from [20].

This is the most commonly used of these new distributed plasma growth methods, being first reported by a consortium of research groups based in Prague in 2011 [19]. In a linear antenna plasma deposition (LAPD) system, 2.45 GHz MW power is delivered into the growth chamber via several pairs of linear coaxial antennas enclosed in quartz envelopes. These linear plasma sources are arranged parallel to one another a few cm above the substrate holder (figure 1). The use of high-frequency MW pulses and low pressures (approx. 1 mbar) increases the plasma electron density to 10^11^ cm^−3^ [20], while pulsing the plasma power at duty cycles of approximately 30% creates ‘on-off’ cycles that allow the gas dynamics to be altered in a transient manner, while minimising the thermal loads [21].

#### (ii) Distributed antenna array MW

**Figure 2.**
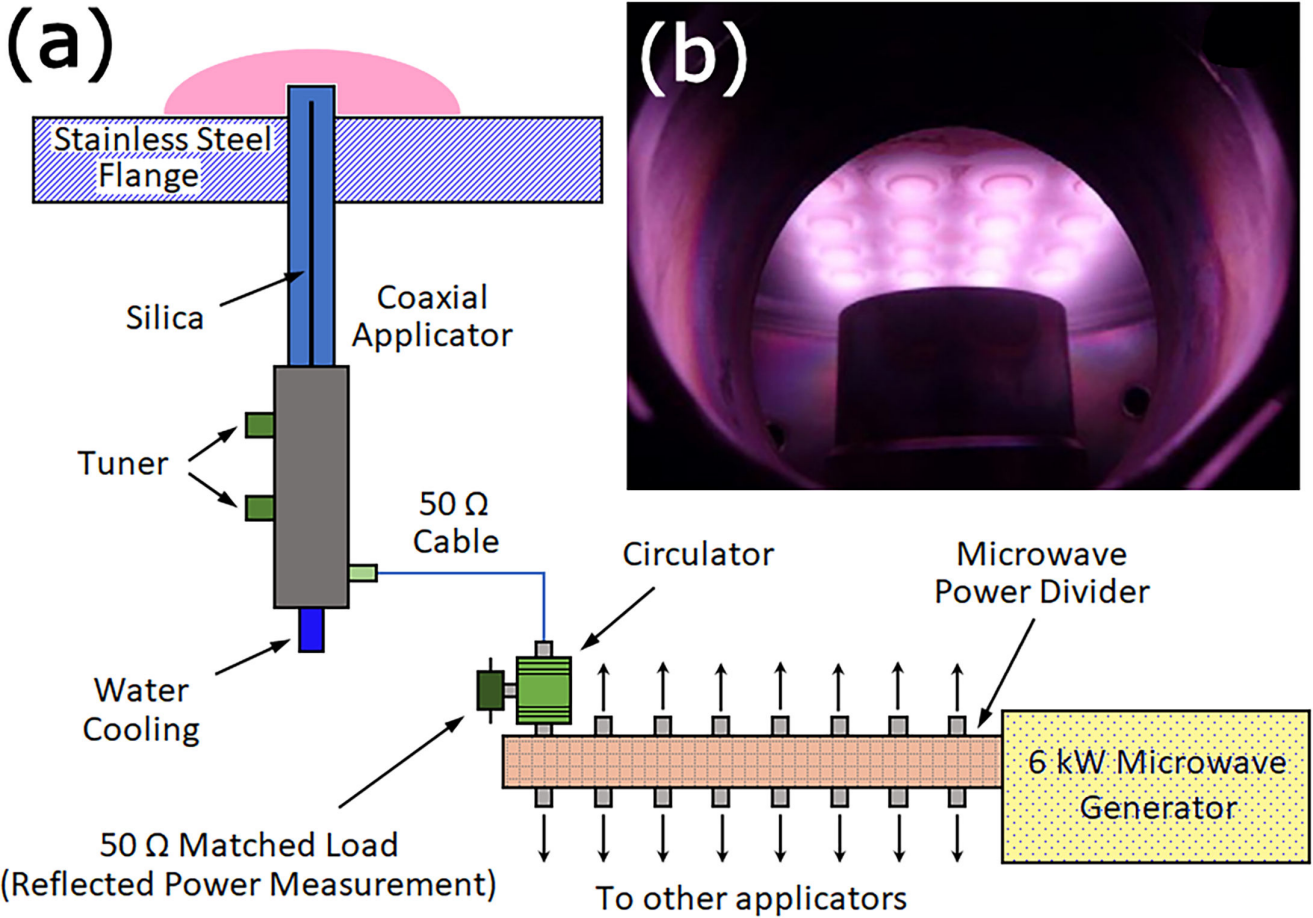
(a) Schematic diagram of a DAAM system, redrawn from [22]. MW power is coupled into a rectangular waveguide and is divided equally between 16 applicators (180 W each), which transport the power to the sources within the chamber using coaxial cables. One of the applicators is shown in more detail on the left; the power passes through a matched load into a tuned antenna protected by a quartz coating, igniting a plasma around each source protruding inside the low-pressure reactor. (b) View of the 16 coaxial ignited plasma sources plasma sources inside the DAAM reactor arranged in a 4 × 4 matrix. Photo reproduced with permission from [22].

A similar deposition process to LAPD, called a distributed antenna array MW (DAAM) system, uses 16 coaxial plasma sources inserted into a square metallic flange in a process chamber and arranged in a 4 × 4 matrix [22]. A 6 kW MW generator is coupled to the system using antennas located in a rectangular waveguide (figure 2). The MW power is divided between the 16 sources, and a discharge is ignited around each source inside the reactor chamber. When the MW power is increased, the plasmas expand and produce a sheet of uniform plasma. The DAAM system allows substrates with sharp edges to be uniformly coated without disturbing the electric field. However, the growth rates and film quality are similar to those deposited by LAPD systems.

#### (iii) Surface wave plasma (SWP)

**Figure 3.**
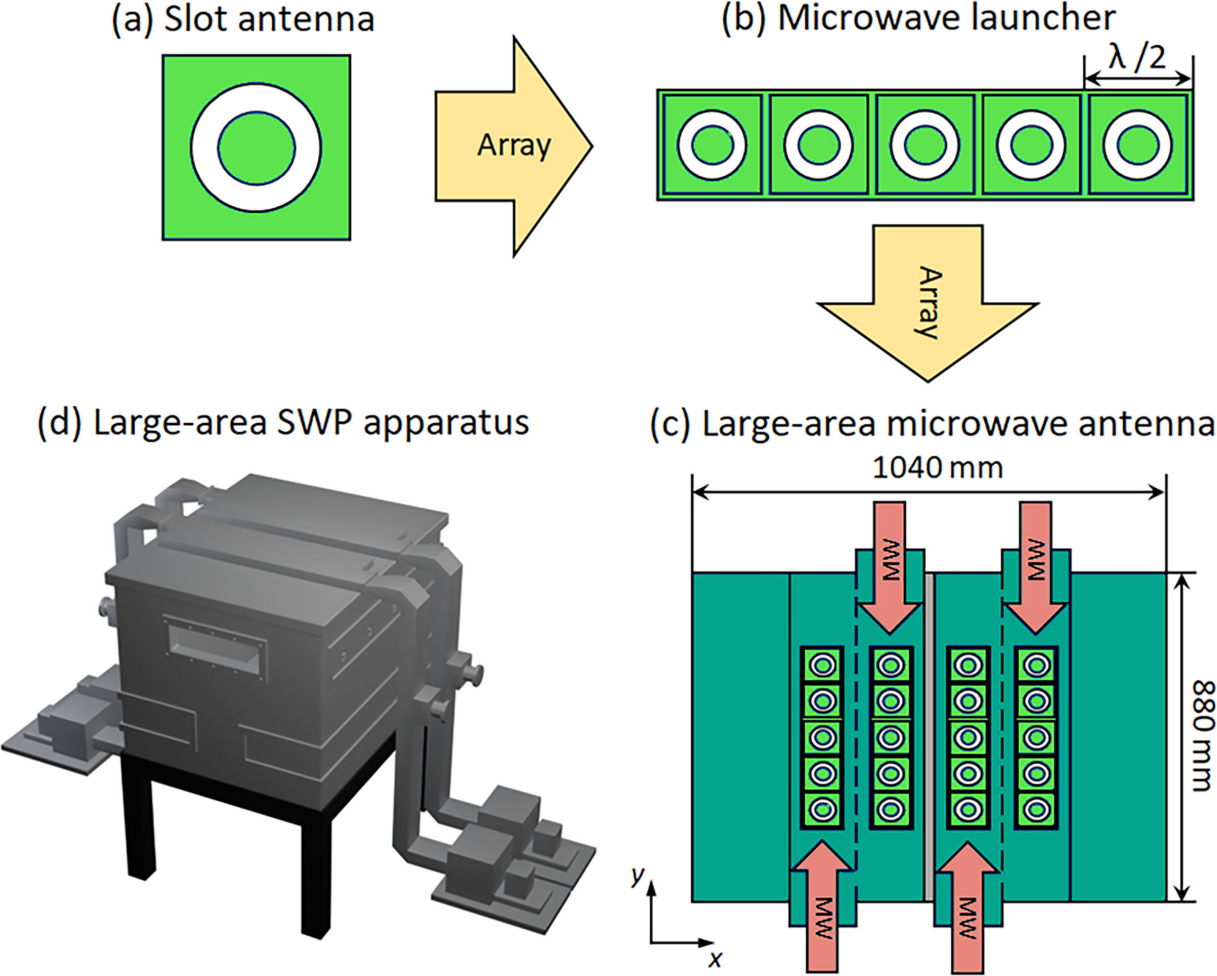
Schematic illustrations of components of a SWP reactor, (a) a slot antenna, (b) a MW launcher with five slots, (c) a large-area MW antenna composed of four launchers and (d) large-area SWP reactor. Figure based upon diagram in [23].

Another new type of system designed for large-area, low-temperature diamond deposition utilizes multi-slot MW antennas and surface wave plasmas (SWPs) [23] (figure 3); SWP reactors use multiple 5 kW, 2.45 GHz MW generators to launch MWs along separate waveguides into which several carefully designed slots have been cut allowing the MWs to access the reactor chamber below. Quartz windows separate the waveguide slots from the vacuum chamber while allowing the MWs to initiate plasmas beneath the slots. Thus, a set of small dense plasmas is formed in a rectangular array allowing substrates of area 600 × 400 mm^2^ to be coated. The substrate stage is water-cooled, allowing nanocrystalline diamond films to be deposited at temperatures as low as 100–500°C.

Because LAPD, DAAM and SWP systems cannot deposit high-quality diamonds at reasonable rates, they will probably remain niche techniques for researchers primarily focused on large-area, low-temperature deposition of smooth films with high mechanical strength and low residual stress.

### (d) MW plasma reactors

**Figure 4.**
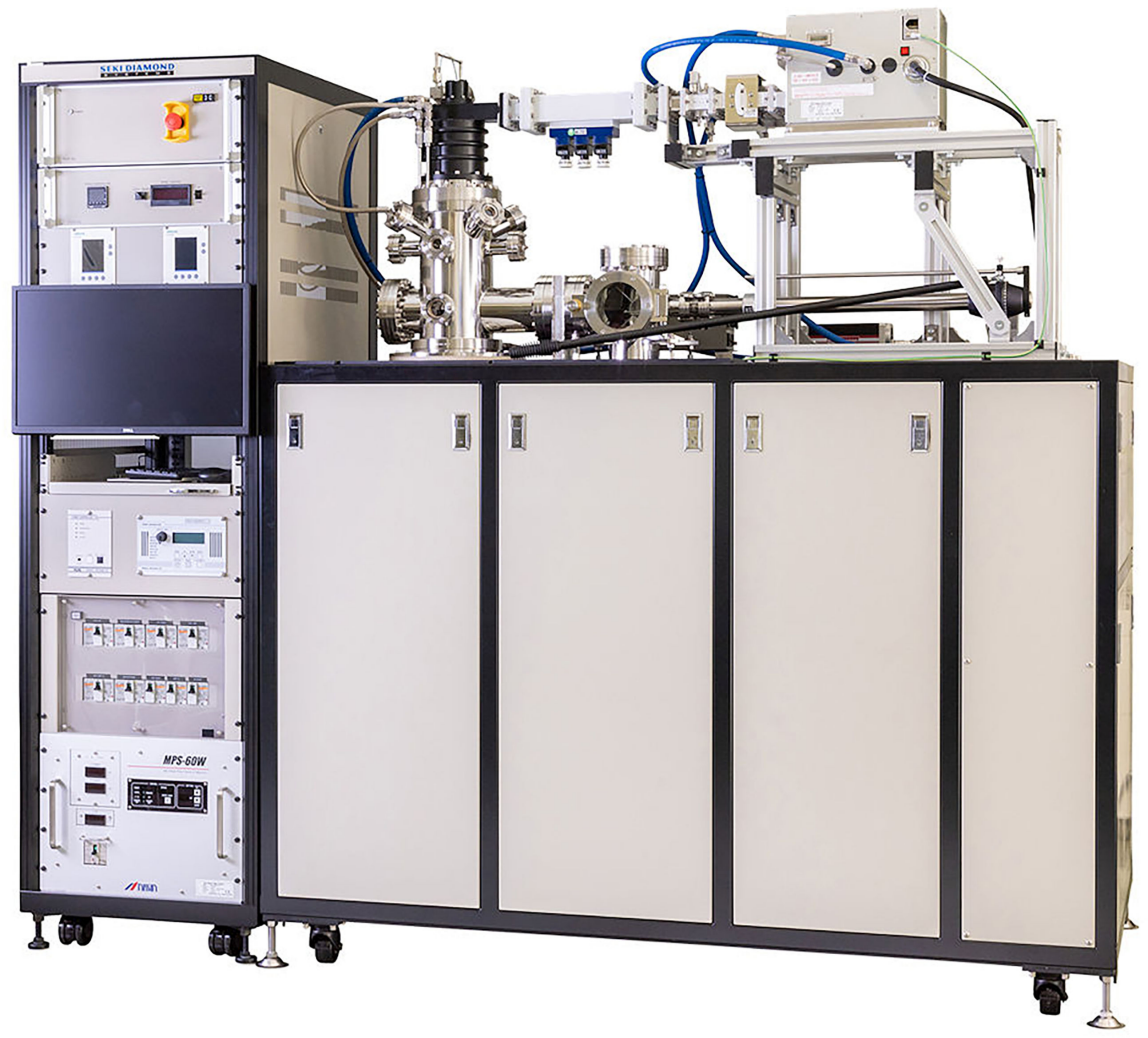
An example of a top-hat MWCVD reactor (Seki model SDS6380). This reactor has power supplies up to 5 kW (at 2.45 GHz) and is capable of depositing polycrystalline diamond films, SCD diamond plates or SCD gemstones at high rates. The chamber is water-cooled, with a substrate platen capable of accommodating samples up to 50 mm in diameter. Photo courtesy of Philippe Bergonzo, Seki Diamond Systems [13].

MW plasmas have now become the standard diamond growth technique of choice for most research groups worldwide, despite their high cost. This is due to their compatibility with existing semiconductor fabrication facilities, reproducibility, ability to deposit polycrystalline and SCD with extremely low levels of unwanted impurities (e.g. residual nitrogen concentrations less than a few parts per billion (ppb)) [24,25], ability to use a wide range of process gas mixtures (especially oxygen-containing gases) and the high growth rates that are possible when using high-power reactors. In the past 20 years, driven mainly by the rapid growth in the lab-grown diamond gemstone industry, the technology behind MWCVD has made huge leaps forward [26]. The preferred design of the MW chamber nowadays is the ‘top-hat’ design, formerly known as the ASTEX-style reactor (figure 4), with the older NIRIM-style reactors [1] rarely used nowadays due to issues with Si contamination from the quartz tube. For larger substrates, ‘clam-shell’ reactors are also popular as they provide easier access to the growth chamber. Although most MW systems currently use 5−6 kW power supplies, the commercial requirements for faster growth rates and larger areas have led to greater-volume MW systems being produced with power supplies up to 100 kW [11]. Because MWCVD requires the deposition chamber to be a resonant cavity for efficient transfer of energy from the MWs to the process gases, the chamber size depends inversely on the frequency of MWs used to generate the plasma. To achieve the necessary power densities over larger areas, the MW frequency is often lowered from 2.45 GHz to 915 MHz, allowing the size of the MW resonant cavity to increase accordingly.

The next important and imminent development in MWCVD technology will be the use of MW solid-state power supplies (SSPS), which, although being slightly less energy efficient than the current magnetron-tube technology, offer a number of advantages. First, for a cost approximately 50% higher than a standard MW magnetron tube, SSPSs provide higher stability (fewer fluctuations in the plasma means fewer growth defects) and a significantly longer lifetime (20–30 years compared to approx. 5 years for a magnetron). However, their main benefit is seen in factories with multiple MWCVD reactors in close proximity. As well as the main MW frequency, magnetron tubes also generate unwanted harmonics that vary from tube to tube. These harmonics can perturb the operation of other nearby magnetrons, causing plasma instabilities and fluctuations in all the plasmas in a CVD factory. To overcome this problem, large CVD facilities use special MW filters or costly uninterruptible power supply circuitry on every reactor to block the interference. In contrast, SSPSs generate only a single pure MW waveform with no harmonics and so do not require an uninterruptible power supply. SSPCs began to enter the market from several manufacturers (e.g. Sairem, Muegge) in 2023, with higher power (6 kW and above) promised to become available within a few years.

MWCVD is by far the most widely used deposition technique in China for diamond *films*. It is used for the growth of the highest-quality diamond films required for electronic, thermal, optical and quantum applications. Chinese lab-grown diamond gemstones currently make up approximately 50% of the worldwide market for synthetic diamond jewellery—and although the high-pressure high-temperature (HPHT) method [11] is the technique of choice for the majority of industrial mass production of SCD in China, MWCVD is preferred for the largest and purest gemstones [11]. At first, the thousands of MWCVD reactors required for this huge endeavour were imported into China from companies such as Plassys (France), iPlas (Germany) and Seki (Japan). But cheaper home-made MWCVD diamond deposition systems were quickly developed by Chinese manufacturers such as Uniplasma (Shenzhen), DMT-Diamond (Xian), Three Three Zero (Chengdu), Heuray Microwave (Chengdu) and Tanfangcheng (Carbon Equations, Shanxi), and these now comprise 50% of the MW growth systems in China [11].

## 4. The chemistry of CVD diamond growth

The ‘standard model’ for diamond growth that was devised in the 1990s [27,28] has stood the test of time and has proven to be an accurate description of the diamond CVD process. This model still underpins our understanding of diamond growth today. The model assumes that because CVD growth temperatures (approx. 1000 K) are below the Debye temperature of diamond (approx. 2240 K), the bulk diamond lattice cannot spontaneously rearrange itself into the more thermodynamically favourable graphite structure. Furthermore, the carbon atoms at each surface are (nearly) all terminated with H, which eliminates any ‘dangling bonds’ that would otherwise cause the surface to restructure into more stable, graphitic structures.

In the growth chamber, the thermal energy from the hot filament or MW energy from the MW power supply dissociates H_2_ into H atoms, which then react with the hydrocarbons present in the gas mixture to create a complex chemical soup of hydrocarbon species. These include neutral molecules and reactive carbon-containing radicals, in particular CH_3_. The H atoms also react with the hydrogens in the C–H bonds that terminate the diamond surface carbon atoms, abstracting the H (to reform H_2_) and in doing so creating surface radical sites. The most likely fate for these surface radicals is that another gas-phase H atom will attach to it, reforming the stable surface. Occasionally, however, a CH_3_ will attach to the surface radical site instead. If there are other nearby surface radical sites, the adsorbed CH_3_ can migrate across the surface until it finds a diamond step-edge, to which it attaches, thereby adding one more carbon to the diamond lattice. Any non-diamond *sp*^2^ carbon species that adsorb are quickly etched back into the gas phase by the H atoms that continually strike the surface. H atoms etch *sp*^3^ carbon approximately 20 times slower than *sp*^2^ carbon, so all non-diamond carbons are swept off the surface, while *sp*^3^ carbons survive long enough to propagate the diamond structure.

Although the standard model is still fundamentally valid, our understanding of the mechanisms involved in diamond CVD has advanced greatly over the past 20 years, as reviewed in detail by Butler *et al*. [29]. This is mainly due to the use of sophisticated *in situ* diagnostics of the growth environment, using various non-invasive spectroscopic techniques such as optical emission [30], cavity ring-down [31] and direct line-of-sight absorption methods [32,33], as well as more intrusive methods such as mass spectrometric sampling from the plasma [34]. Studies such as these provided key quantitative data about the spatial distribution and concentrations of many of the gas-phase species within hot-filament, MW-plasma and DC arc-jet reactors. These values enabled the development of accurate three-dimensional (3D) models of the chemical processes and gas dynamics in the growth chamber under CVD conditions, which, in turn, made it possible to estimate similar data for the species that are less amenable to spectroscopic analysis (such as the key CH_3_ radical).

**Figure 5.**
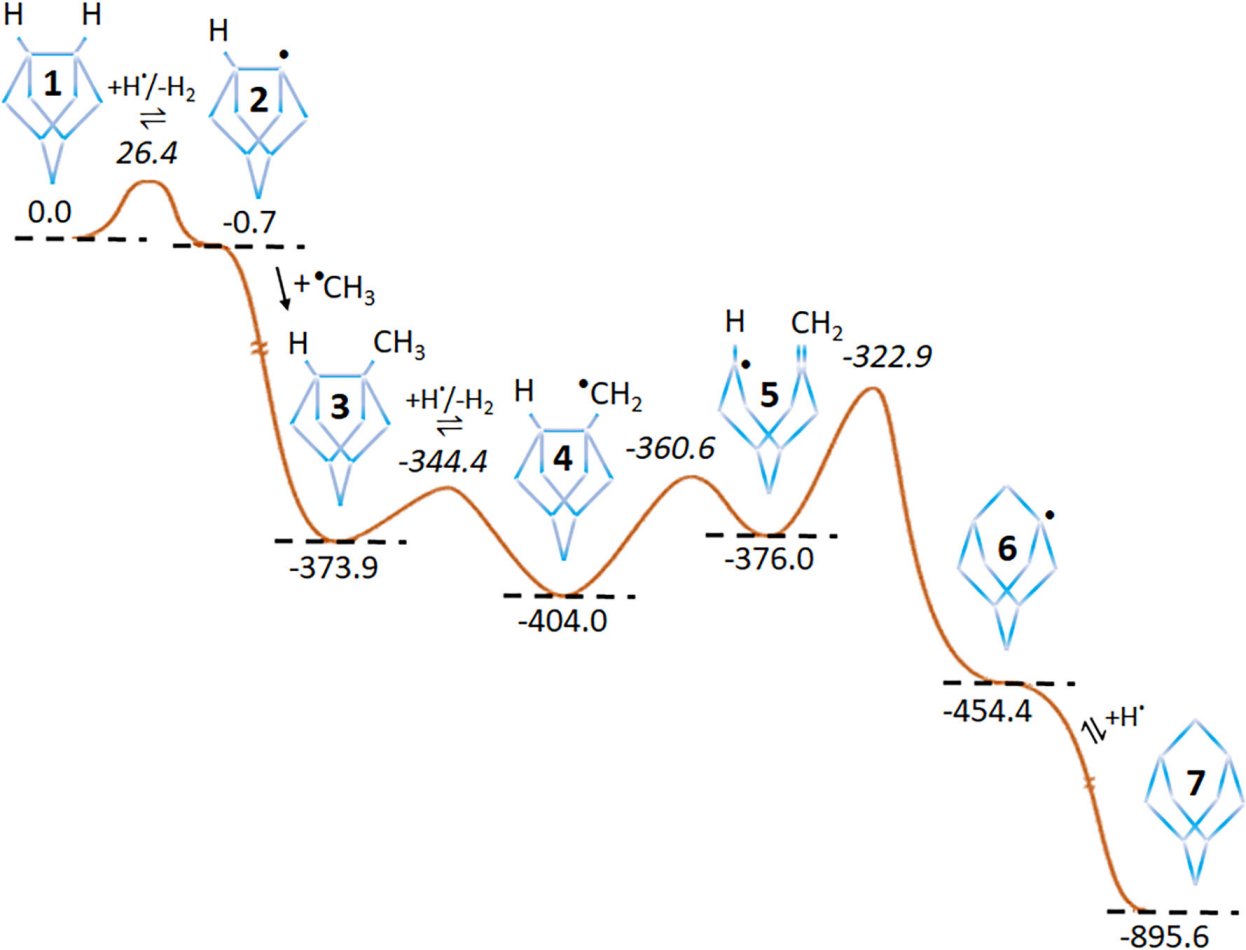
Series of elementary steps by which an incident CH_3_ radical can insert into a C−C dimer bond on the C(100):H (2 × 1) surface. The respective minima (normal font) and transition state (in italics) energies are in kJ mol^−1^ relative to that of structure 1. The dots indicate the location of a radical group, i.e. a ‘dangling bond’ with an unbonded electron. Redrawn using data in [29].

**Figure 6.**
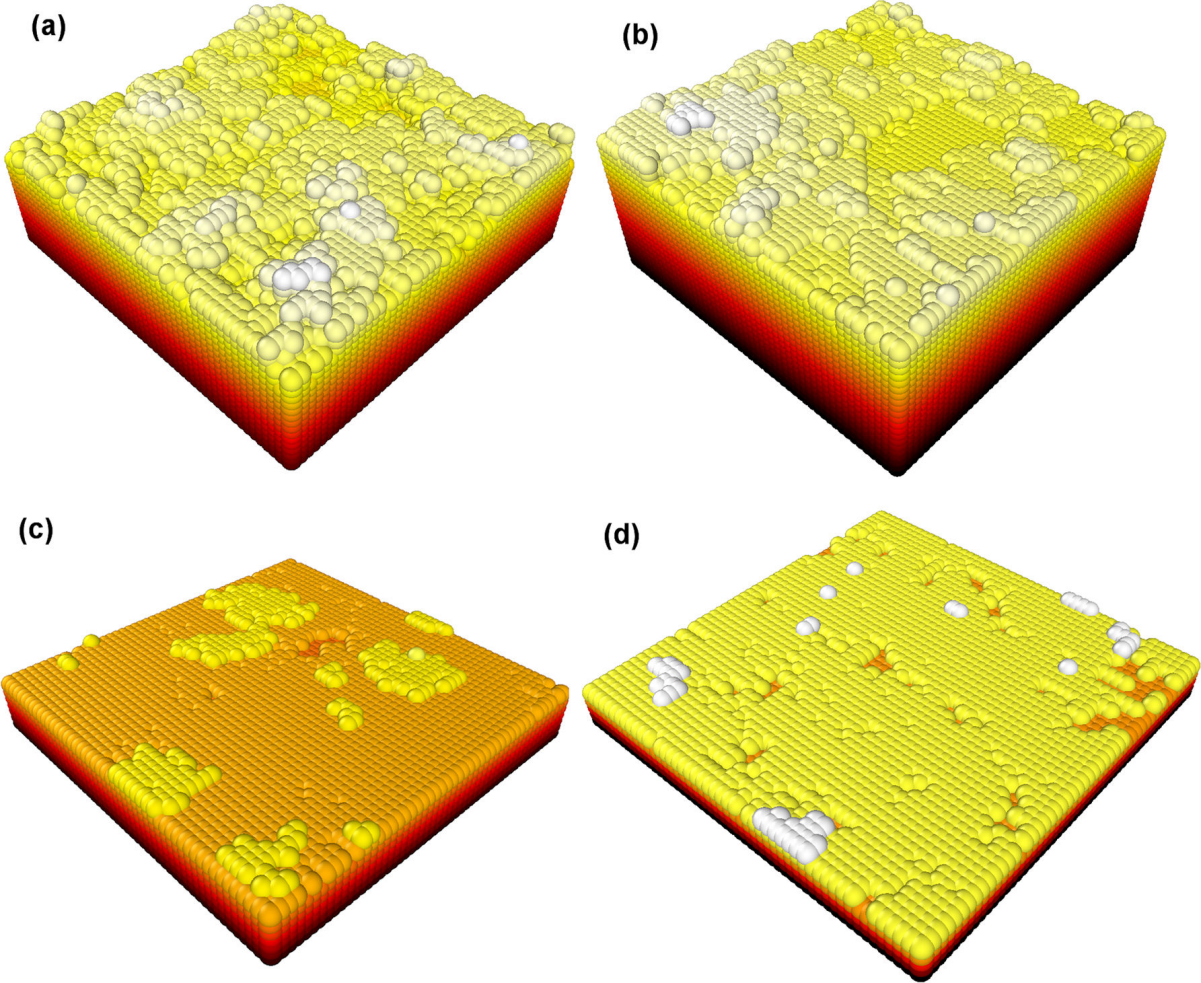
3D projections of the surfaces generated by a 3D kMC model representing the different types of diamond growth resulting from different process conditions. More details of these diamond film varieties can be found in §5. (a) UNCD, with no crystal faces visible as a result of continual random renucleation. (b) Nanocrystalline diamond (NCD), with some small terraces appearing (i.e. nanocrystallites) as a result of a layer-by-layer growth coupled with renucleation. (c) Microcrystalline diamond (MCD), where layer-by-layer growth dominates leading to large micron-sized faceted crystallites. (d) SCD, where growth is now entirely by layer-by-layer growth. The layers have been coloured to make it easy to distinguish individual layers. Reproduced with permission from [42] under the Creative Commons CC BY 3.0 licence.

In addition, individual gas-phase and gas-surface reactions, particularly for the diamond C(100):H (2 × 1) surface, were simulated at the molecular level by quantum-mechanical codes such as density functional theory (DFT) [35]. These enabled potential reaction pathways to be studied (figure 5), determination of the relative stability of surface adsorbates [36], the role of dopants in the growth process [37–39] and the function of different reactants and mechanisms in growth [40]. The data from these various experimental measurements and computer simulations were then combined into macro-scale computer models of the whole CVD process, enabling processes such as surface migration, nucleation and step-edge growth to be predicted [41]. More recent kinetic Monte Carlo (kMC) models [42,43] (figure 6) have predicted growth rates, crystallite morphology, etch-pits and the role played by an immobile ‘critical nucleus’ in the initiation of a new diamond layer.

Although the standard growth model is well accepted, there are still some aspects of the mechanism that remain controversial or unresolved. A detailed discussion of two of these issues is given in the electronic supplementary material, but a summary of the important points is provided in the next two sections.

### (a) The C_2_ controversy

The first controversial issue concerns the importance, or otherwise, of the C_2_ radical as a growth species. Key to the standard model is the idea that the main growth species are CH*_x_* (*x* = 0–3) and, in particular, the methyl radical, CH_3_ [28], which is supported by a wide range of direct and indirect evidence. These ideas were challenged, however, in 1994, when a paper reported that diamond films could be grown in a MW plasma using a mixture of Ar and vapourised C_60_, i.e. with apparently no H present [44]. The new type of diamond film, termed ultrananocrystalline diamond (UNCD), consisted of approximately 4 nm diamond crystallites, and it was suggested that with allegedly no H present, a different growth mechanism must be operating to that for normal CVD diamond. In the absence of CH_3_, the C_2_ radical was suggested as the growth species. Indirect evidence was presented for this contentious view in that the plasma was bright green in colour due to emission from the bright Swan bands of the C_2_ radical [45], in contrast to the lilac colour of traditional CH_4_/H_2_ plasmas, while DFT calculations showed that C_2_ radicals were able to insert directly into surface dimers [46].

However, in the few years following these first reports of ‘H-free diamond growth’, no research groups were able to reproduce the results using the Ar/C_60_ recipe. In all cases, UNCD films were successfully deposited *only* when trace amounts of H_2_ were added to the Ar/C_60_ gas mixture, or alternatively using Ar/CH_4_ mixtures that produce H atoms upon fragmentation—otherwise the films were graphitic. This suggested that maybe there were trace amounts of H_2_ from an unknown source present in the original Ar/C_60_ experiments. Cavity ring-down spectroscopy was used to measure the absolute concentration of C_2_ in the plasma during UNCD growth, and this was found *not* to correlate with the film growth rate [47]. Experimental and modelling results of the gas-phase concentrations during UNCD growth showed that, although the concentration of C_2_ was high in the centre of the plasma (and gave rise to the bright green emission), its concentration fell by many orders of magnitude close to the cooler diamond surface [48] and was insufficient to account for the observed growth rate.

Nowadays, the C_2_ growth mechanism has largely been discounted as a viable mechanism for UNCD growth—or for the growth of any form of diamond [49]—although it is still sometimes cited in papers! However, the role of C_2_ (and the more abundant C_2_H) in renucleation and defect formation is still very much a possibility.

### (b) The nitrogen effect

**Figure 7.**
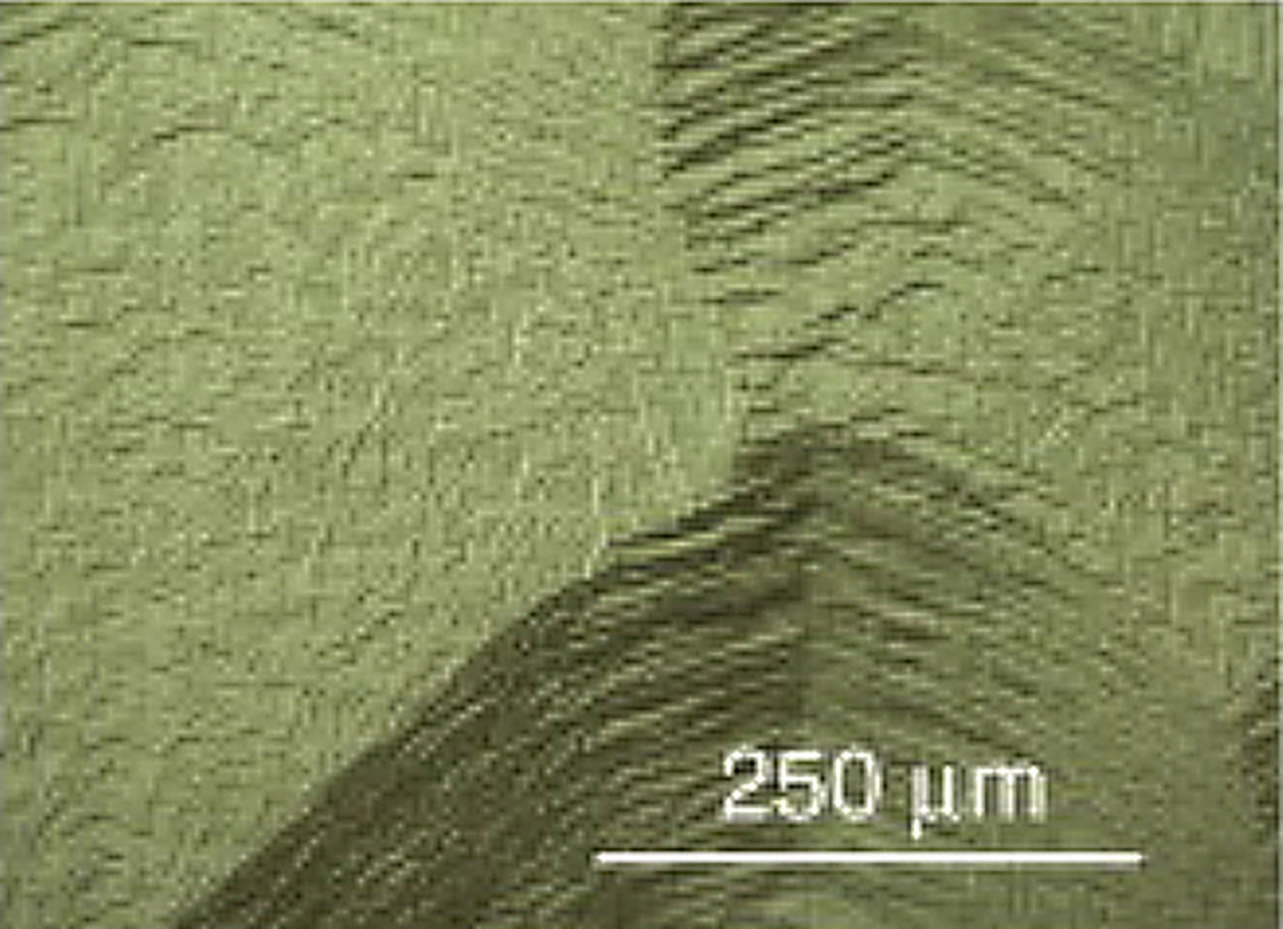
SEM image showing macroscopic step-bunching on the surface of an SCD sample grown using nitrogen-containing gas mixtures. Reprinted with permission from [51].

The second controversial issue regarding diamond growth involves the so-called catalytic role played by nitrogen. Many studies have shown that even *trace* amounts of N_2_ in the process gas mixture significantly increase the growth rate from MW-activated CH_4_/H_2_ gas mixtures, sometimes by a factor of 10 or more [50,51]. The morphology of the growing diamond surface is also modified by the presence of nitrogen in the gas mixture, which encourages the preferential formation of flat, square {100}-faceted crystals in the case of polycrystalline diamond films. Excess nitrogen in the process gas mixture, however, produces smaller and less well-oriented surface facets. With further increases in gas-phase nitrogen concentration, the films become nanocrystalline or even graphitic [52,53]. For homoepitaxial growth of SCD, nitrogen in the input gas mixture has been shown to promote macroscopic step-bunching (as shown in figure 7) and disrupt growth on all but the {100} face of an SCD seed [54].

To avoid these issues, in the interests of process control and reproducibility, diamond researchers are now far more careful to control unwanted N_2_ (and other) impurities in the gas mixture. It is now common to add high-vacuum turbopumps to CVD reactors to reduce the chamber base pressure to less than 10^–6^ mbar prior to diamond growth, along with the use of process gases with extremely high purity. Such careful control has allowed electronic-grade SCD films to be fabricated which contain N concentrations of less than 0.1 ppb [24].

This so-called ‘nitrogen effect’—the addition of tiny amounts of N_2_ to the process gas mixture causing dramatic changes to the growth rate and morphology—is still not fully understood. Indeed, nitrogen has sometimes been described as acting as a ‘catalyst’ in the growth, due to a small concentration causing a large effect, but this term is not strictly correct as the gas-phase nitrogen is not unchanged in the reaction—some small fraction ends up incorporated in the film. So how does the usually inert N_2_ molecule cause such huge effects?

To answer this, a combination of experimental studies (such as laser absorption and OES) and computer modelling of MW-activated N_2_/H_2_ and NH_3_/H_2_ plasmas revealed that the hot plasma environment could create excited-state N_2_ species [55,56]. Some of these excited-state N_2_ molecules have sufficient energy that their reactions with other gas-phase species or with the diamond surface break the strong N_2_ triple bond. Thus, unreactive N_2_ is converted to more reactive N-containing radical species such as N atoms and NH, NH_2_ and CN radicals, which can now participate in the diamond CVD process.

Theory suggests that these reactive N-containing species should be able to insert into a C−C dimer bond on the C(100):H (2 × 1) surface via a ring-opening/ring-closing reaction mechanism [57] analogous to that shown earlier in figure 5 for the case of CH_3_ addition. These reactions provide viable routes for N incorporation into substitutional sites in the diamond lattice, but they do not explain the unusually large effect that N has on growth rates and morphology.

Theoretical calculations have been used to investigate a number of possible mechanisms. The first suggests that adsorbed NH species at different step edges on the C(100):H (2 × 1) surface enhance the binding of gas-phase CH_3_/CH_2_ groups at these locations [58]. A second suggestion is that the additional electron density provided by a buried near-surface N atom weakens any nearby surface C−H bonds. This enhances the rate of the H-abstraction step that creates the surface radical site necessary for CH_3_ radical addition [59]. Other mechanisms explain the apparent ‘catalytic’ nitrogen effect as a consequence of a layer-by-layer growth mechanism growth model in which the rate-limiting step is the nucleation of a new layer. Initiation of a new layer requires the creation of a ‘critical nucleus’—an immobile surface feature with a low etch rate, which acts as a starting point for subsequent rapid lateral growth. Indeed, Monte Carlo models using step-flow growth conditions that include such critical nuclei have demonstrated tenfold enhanced growth rates [42,60]. Butler & Oleynik [61] suggested that adsorbed CN might act as just such a critical nucleus because it cannot undergo the *β*-scission reaction responsible for trimming longer-chained hydrocarbons from the diamond surface (see electronic supplementary material, fig. S1). Hence, it only takes a single CN adsorbate across an entire surface composed of billions of carbon atoms to initiate a new layer, which explains the apparent catalytic effect. A similar mechanism involving the critical nucleus being a lone C–N dimer sitting among the thousands of C–C dimers on the flat (100) surface was recently proposed by Oberg *et al*. [62]. It remains unclear which of the many proposed mechanisms is responsible for the nitrogen effect, or whether it is a combination of several of them.

### (c) Remaining issues in CVD diamond growth

Despite the continued success of the standard model of diamond growth and the updates and improvements made to it over the past 20 years, there are still a number of details about the growth mechanism that remain elusive. Unfortunately, research has almost stopped in this fundamental area, partly as a result of the community and funding agencies believing (incorrectly) that the growth mechanism was essentially ‘solved’, but also because, after 20 years of research, funding agencies are now focusing on applications of diamond technology that can produce demonstrable commercial products, rather than fundamental understanding. However, in many cases, it is the ignorance of the basic science that is inhibiting progress with many applications. Some of the unsolved problems are as follows:

— What is the role in growth (if any) of larger hydrocarbon species, such as C_2_ or C_2_H? If their role is detrimental to growth rate or diamond purity, can the plasma conditions be modified to reduce or eliminate these species?
— What causes renucleation? It is clear that as the concentration of C > 1 hydrocarbons in the gas phase increases (e.g. by increasing the CH_4_:H_2_ ratio), the rate of renucleation increases, leading to twinning and smaller crystallites. But exactly which species cause renucleation, and by what mechanism, is still not known.
— The reaction rate for a CH_3_ radical attaching to a surface radical site is limited by a temperature-dependent sticking factor, *P*. The value of *P* results from a combination of factors that reduce the reaction probability, such as a geometrical factor (*g*) due to unfavourable collision orientation and a steric-electronic factor (*s*), arising from the electron spin selection rules involved in bond formation. Also, only a fraction, *F*, of the total surface radical sites will be accessible for adsorption, and the value of *F* will depend upon the surface temperature and local H-atom concentrations. Thus, *p* = *g* × *s × F*, and the three factors can be estimated for different growth conditions [63,64]. For example, electronic-spin statistics show that for CH_3_, on average, three collisions out of four will be on the triplet surface and will not lead to reaction at the high temperatures of diamond CVD [65], i.e. *s* ~ 0.25, while typically *F* ~ 0.5 for CVD conditions [63]. The value for *g*, however, remains little more than a ‘best guess’. As is clear, the uncertainties in these estimates are large, leading to predicted growth rates from computer simulations that can differ from experiment by an order of magnitude. Molecular-dynamics calculations of the reaction process may provide more accurate, or at least more justifiable values [66,67], but nevertheless, the value of *P* and its variation with growth conditions and surface orientation remain key inputs to various growth models that urgently need refining.
— Although many macroscopic simulations of diamond CVD confirm the role of surface migration in step-edge growth and surface smoothing [41,42], the exact mechanism by which this happens remains somewhat vague. In particular, the rates with which adsorbed CH_2_ ‘bridge’ species migrate across adjacent dimer reconstructions, or into vacant trough sites, or down atomic steps, are all unknown and yet are key to the quantitative modelling of diamond CVD.
— Trace amounts of nitrogen in the gas phase are known to significantly increase the growth rate and change the surface morphology. There are many suggested mechanisms for this (see earlier), but none have yet been confirmed experimentally.
— Are there strategies, other than simply increasing MW power, that might be used to improve growth rates or uniformity, such as addition of trace gases (O_2_, halogens, noble gases, D_2_), or improved gas injection configurations [68] or tuning the substrate position [69]?

## 5. The CVD diamond film

When diamond is grown *homo*epitaxially [24] on to an existing SCD seed crystal (which can be a natural diamond or one grown by CVD or HPHT methods), then the product is an enlarged SCD that can either be cut and polished into a gemstone (which is the basis for the flourishing CVD diamond gemstone market [2]) or laser cut into flat SCD substrates usually a few millimetre in size, suitable for advanced applications or further seeds.

**Figure 8.**
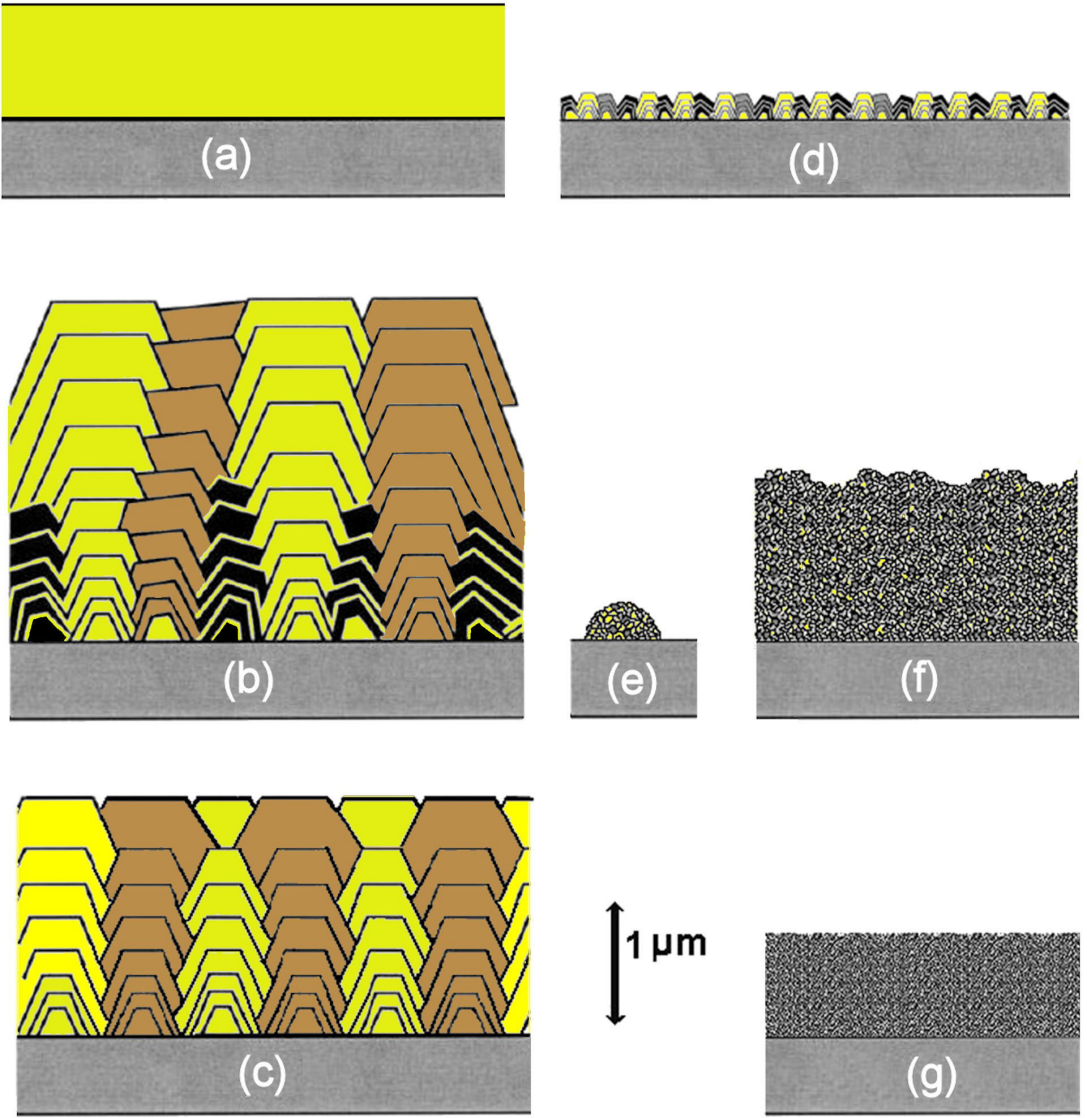
Schematic representation of various types of CVD diamond film deposited on to a substrate (grey). The colours yellow, brown and black are used to allow the reader to discriminate between different diamond crystallites (grains) and their growth evolution. (a) SCD film grown epitaxially on an SCD substrate. (b) MCD columnar growth from randomly located nuclei, where the slowest growth face determines the overall film texture, in this case (100). (c) Highly oriented textured MCD obtained following special nucleation procedures, such as BEN (see §7). (d) Faceted NCD, which is really just thin MCD with high nucleation density. (e) ‘Cauliflower’ (or ‘ballas’) NCD before it has coalesced into a continuous film. (f) Cauliflower NCD film. (g) UNCD. Computer simulations of some of these diamond-film types were shown previously in figure 6.

In contrast, *hetero*epitaxial growth (diamond grown on to a non-diamond substrate) begins from numerous individual isolated nucleation sites, e.g. from microdiamond or nanodiamond seed crystals scattered on the surface, or surface defects such as scratches, impurities or dislocations [6]. The individual diamond nuclei enlarge as gas-phase carbon species adsorb on to them, and new carbon atoms add to the diamond lattice. The nuclei grow in three dimensions until they meet their immediate neighbours, whereupon they fuse together to form a coalesced (or closed) two-dimensional (2D) film that then continues to grow normally to the surface. The resulting material will usually be polycrystalline, composed of almost pure diamond crystallites (or grains) joined together by grain boundaries that contain varying amounts of impurities and non-diamond carbon. The morphology and the average crystallite size, and hence the resulting film properties, can be tuned [70] simply by choosing the appropriate process conditions [71], as shown in figure 8.

Diamond films are usually grouped into categories depending on their crystallite size and morphology.

### (a) SCD

Figure 8a is a near-perfect diamond crystal lattice with sizes that can approach several centimetres. It is usually deposited by templating on to an existing SCD seed, which can be later removed by laser cutting. Minimizing any remaining defects in the SCD layer (dislocation density, impurities, vacancies, etc.) is currently the goal for both the gemstone industry and for electronic device applications, as in both cases, ultra-high-purity SCD is the ‘billion-dollar prize’.

### (b) Microcrystalline diamond

These films (figure 8b) exhibit faceted crystallites of size 0.5 µm to a few 100 µm, with a columnar growth structure that produces anisotropic properties that vary with film thickness. The grain boundaries between crystallites, which contain non-diamond carbon, are typically only a few angstroms thick. The crystallites in microcrystalline diamond (MCD) films are usually randomly oriented, as a result of the nucleation being from seeds or surface scratches that were themselves randomly located and oriented. As the film grows, the fastest growing surface (110) grows out via the van der Drift competitive growth model [72], leaving only the slowest growing facets, (100) or (111), visible at the film surface. The ratio of square (100) to triangular (111) facets in the film depends upon the growth conditions and is quantified by the α-parameter [73]. So-called ‘textured’ MCD films can be made using seeds that are arranged to be oriented all in the same direction, or by using specialized seeding nucleation strategies such as BEN [74] (see §7). These films grow preferentially in the (100) orientation, and as the growth islands merge, the resulting continuous film can appear as near single-crystal over large areas (figure 8c).

### (c) Nanocrystalline diamond

These films [75] contain crystallites ranging in size from approximately 10 to 100 nm. Unfortunately, there are two types of diamond film that are often labelled ‘NCD’ (nanocrystalline diamond) in the literature. Diamond films grown with high nucleation density but for short periods of time, such that the films are less than 1 μm thick, have a facetted morphology and grain boundaries, just like MCD, except the crystallites are 10−100 times smaller. These NCD films have almost identical properties to MCD, except their ratio of grain boundaries to crystallites is larger due to their smaller size (figure 8d). Alternatively, NCD films can also be made by increasing the hydrocarbon concentration in the CVD gas mixture. This changes the growth chemistry, increasing the rate at which renucleation occurs and promoting the growth of secondary crystallites as ‘off shoots’ of the original nucleus. The greater the C:H ratio in the gas mixture, the higher the rate of secondary nucleation, resulting in a larger number of smaller crystals. This type of diamond growth leads to more rounded crystallites, which form into hemispherical agglomerates (figure 8e), sometimes referred to as ‘cauliflower’ or ‘ballas’ diamond (figure 8f). The grain boundaries in cauliflower diamond films can be several nanometres thick and contain *sp*, *sp*^2^ and *sp*^3^ hybridised carbon bonded in variety of configurations, along with impurities such as H and N. Although NCD films have properties that are generally inferior to MCD, their nanometre-scale surface roughness often makes them useful as wear-resistant protecting coatings in mechanical applications [76].

### (d) UNCD

UNCD films [77,78] have crystallite sizes less than 10 nm embedded in an *sp*^2^-carbon matrix, and although their properties are less extreme compared to MCD and NCD, they have the advantage of being smooth on the nanometre scale (figure 8g) so that they can be used as vacuum pump seals [79] or medical applications [80]. UNCD is deposited using gas chemistries that have a much lower hydrogen concentration than for standard CVD conditions, for example, 1% CH_4_ in Ar, where the H:C ratio is only 4 compared to greater than 200 in standard 1% CH_4_/H_2_ mixtures. The grain boundaries in these films can be several nanometres wide, making them almost the same size as the diamond crystallites. These boundaries (especially when doped with N [81]) can form electrically conducting pathways through the film, allowing them to be used in electronic device applications [82].

## 6. The substrate material

A major goal in the field of diamond CVD is the lowering of substrate temperatures required for growth, as this would permit the use of a much wider range of substrate materials of industrial importance. Das & Singh recently published a comprehensive review [83] of the many attempts to deposit CVD diamonds at lower temperatures. These included methods such as varying the composition of the gas mixture (in particular by addition of O-containing gases or halogens) or by alteration of the processing conditions, such as by coating the substrate with another material, pulse modulation of the MW plasma or cyclic modulation of the CH_4_ flowrate. Many of these techniques succeeded in lowering the deposition temperature by 100–200°C, but usually at the cost of greatly reduced growth rates, poorer quality diamonds and/or significantly added complexity to the CVD process. Most researchers now grudgingly acknowledge that for high-quality diamonds, there is no option but to deposit at temperatures greater than 700°C and accept the substrate limitations this entails.

The major problem associated with such high-temperature growth is that many common industrial materials, such as plastics, glass, semiconductors (GaAs, CdS and CdSe) and metals (Al, Zn, Sn, Pb, Mg and many of their alloys) have melting points lower than the diamond deposition temperature and so cannot be used as substrates. Many remaining materials (e.g. Ti, Fe (including steels [84]), Ni, Co and Cr) either have a solid-state solubility for carbon that is too high at standard CVD process temperatures, such that most of the deposited carbon reacts to form a metal carbide rather than a diamond film, or are unreactive towards carbon (Cu and Ge), such that the deposited diamond film grows but does not adhere to the substrate. Moreover, some materials when heated to CVD diamond temperatures ( greater than 600°C), notably Fe, Co and Ni, catalyse the conversion of *sp*^3^ carbon into *sp*^2^ phases, promoting graphitization over diamond deposition.

Of the remaining materials, thermal expansion mismatch is then often the major problem. The substrate expands at the high growth temperature, and the diamond film then grows upon this expanded substrate. The sample is then cooled back to room temperature, whereupon the substrate contracts back to its normal size while the attached diamond film—with a much lower linear coefficient of thermal expansion (CTE)—contracts significantly less. The stresses cause the sample to bow with the diamond side outermost, the diamond film to crack or delaminate or the entire sample to fracture into pieces, depending on the strength of the adhesion between the substrate and the diamond layer.

These problems highlight another of the ongoing problems with diamond CVD technology, which has been around since the 1990s—there remain only a few materials upon which high-quality diamond films can be deposited. Apart from the diamond itself, silicon remains the substrate of choice due to its high melting point, low cost, availability as flat, polished wafers, low(ish) CTE (only about three times higher than that of diamond compared to those for most metals which are typically 10−20 times higher) and propensity to form a thin (a few nanometres) carbide to enable the diamond layer to adhere. Other ‘diamond-friendly’ materials are given below.

### (a) Oxides and nitrides

It is possible to grow thin layers (a few micrometres) of diamond on to thin (a few micrometres) layers of SiO_2_ and Si_3_N_4_ on a Si substrate. But if the diamond layer or the oxide/nitride layer becomes too thick (e.g. for *bulk* Si_3_N_4_ or SiO_2_, or quartz), the CTE mismatch often causes the diamond film to delaminate. The same problem occurs with other oxides and nitrides, such as Al_2_O_3_ [85], sapphire [86], Ga_2_O_3_ [87], MgO [88] and TiO_2_ [89], which in some cases can be minimized by optimizing the growth conditions. In contrast, many metal oxides can be deposited on to diamonds for use as gate oxides [90], heterojunctions [91] or as surface transfer doping layers [92] (see §12b(ii)).

Diamond can be successfully deposited on to hexagonal [93] and cubic boron nitride [94], although, as for oxides, more work has been reported for the flipped approach—deposition of boron nitride on to diamond [95]. GaN has been of particular interest as a substrate for diamond growth due to its use in high-power electronic devices [96], such as base stations for transmitters used in 5G mobile phone networks [97]. A diamond heat-spreader thermally bonded to a hot GaN device would rapidly dissipate the heat to a remote heat sink or cooling system, enabling the GaN device to operate at higher power loads with a longer lifetime [98]. The room-temperature CTE of GaN is 28% larger than that of diamond [99]; however, the bigger problem is the diamond growth chemistry. Although its normal melting point is approximately 2500°C, GaN reacts with hydrogen at temperatures around only 800°C, decomposing to volatile NH_3_, N_2_ and GaH_3_ resulting in etching of the GaN [100]. Moreover, the interface between GaN and diamond is rather weak because Ga does not readily form a carbide. Thus, the as-grown diamond adheres to the GaN mainly via weak non-covalent interactions, rather than strong covalent bonds, making it prone to delamination. Reasonable quality diamond films have been deposited on to GaN at lower temperatures or using a thin SiC or SiN barrier layer [101], but these tend to have poor adhesion and poor thermal conduction across the GaN/diamond interface—which is a crucial failure for heat spreading applications [102]. Recent work has shown that thick diamond layers can be grown on to AlN [103], which can also be used as a relatively high-thermal-conductivity barrier layer on GaN.

### (b) Metals

For pure metals, diamond grows well on Mo, W, Re and Ir, with the latter being used for growth of heteroepitaxial SCD layers (see §7). Beryllium has also been reported to be a suitable metallic substrate for diamond growth for films less than 30 μm thick, so long as the growth temperatures exceed approximately 750°C required to convert the native oxide (BeO) into carbide (Be_2_C) [104]. For Cu, its inability to form a carbide combined with its very high CTE has meant that diamond films that grow on its surface delaminate upon cooling. This has led to it being used to fabricate freestanding diamond plates [105].

### (c) Carbides

Diamond also grows well on many carbides, in particular, WC [106] and SiC [107], due to the fact they are already saturated with C. This means none of the depositing carbon is lost by dissolving into the substrate or reaction to form a carbide. Their low CTE values also help prevent delamination.

### (d) Silicides

Very little work has been reported about deposition on to these materials, but some silicides have been shown to meet the criteria for diamond growth, such as Ni_3_Si [108] and CoSi_2_ [109], but less so on Fe*_x_*Si (*x* = 0.5, 1, 3) [110].

### (e) Graphite

Diamond growth on to graphitic or *sp*^2^-carbon-rich materials is tricky because the CVD conditions are designed to etch *sp*^2^ carbon. Diamond has been deposited on to bulk graphite [111,112], but this usually requires careful control of the CVD conditions to reduce the possibility of etching. The trick here, at the start of the deposition process, is to use a very high seeding density—almost a continuous monolayer—together with low deposition temperatures and high CH_4_/H_2_ ratios. These conditions aim to make the initial growth rate exceed the graphitic etching rate, thereby rapidly creating a protective continuous layer of diamond over the whole surface, which protects the graphitic substrate from the hydrogen atmosphere before it has a chance to etch. Once this protective coating is continuous, the CVD conditions can revert to normal, to grow higher-quality diamonds at higher rates. More recently, similar growth techniques have been used to diamond coat carbon fibres [113], carbon-fibre composites [114] and carbon nanotubes [115].

Depositing diamonds at much lower temperatures (200°C) has recently become possible using LAPD, DAAM or SWP systems (see §3). These systems have very low deposition rates, but the films they deposit have high *sp*^2^ carbon content and are best described as NCD or even UNCD. Nevertheless, they are hard, smooth diamond coatings that can be deposited as thin (less than 10 nm) layers on many substrates that are impossible to coat with conventional high-temperature CVD. These substrates include glass [23,116], fibre optics [117], plastic [118] and infrared optics [119].

## 7. Nucleation

For heteroepitaxial growth of diamond on to non-diamond substrates, the long initial induction period before which diamond starts to grow can be significantly reduced using a suitable pre-treatment prior to deposition (see the excellent review of nucleation by Mandal [120]). Many different types of pre-treatments have been reported to realize high uniform nucleation density leading to good quality diamond film growth. The list includes mechanical abrasion/scratching, seeding, electrical biasing, covering/coating with Fe or amorphous C, ion implantation, pulsed laser irradiation and carburization, all reviewed in detail in [83]. Mechanical abrasion and seeding were well-known in the 1990s [1]. But many of the other techniques in that list are used rarely, or only for specialized substrates. Perhaps the most important advance relevant for diamond nucleation in the last 20 years has been the discovery and commercial production of detonation nanodiamond (DND) [121].

DND particles typically have sizes 2−10 nm [122], and their most common production method is via detonation of explosives (such as trinitrotoluene and hexogen) in an inert atmosphere or in water/ice, inside a steel chamber [123]. The detonation produces a supersonic shockwave within which the prevailing high pressures and temperatures are sufficient to crystallize carbon into diamond. However, after the transient shockwave passes, the conditions revert to less extreme pressures and temperatures, favouring other forms of carbon. Thus, the explosion produces a mixture of nanodiamond particles, soot and other *sp*^2^ carbon material.

The powdery mixture of detonation products is cleaned with various acids and reagents to remove unwanted metallic impurities and soot, and the diamond component is extracted. The resulting DND material is now commercially available from many suppliers worldwide (see electronic supplementary material, table S1) as a powder or as a suspension in water and is currently produced at a rate of several tons per year and sold for as little as $100/kg. Unfortunately, the DND particles tend to fuse into aggregates approximately 100 nm in size, and thus the as-supplied material usually requires de-aggregation before subsequent processing. This can be achieved in many ways, including ball milling, pulverization, high-power sonication, acid treatments, controlled heating in O_2_ or H_2_ or combinations of these methods [124]. The DND particles that are finally obtained are often described as having a diamond core surrounded by a (partially) graphitic or fullerene-like shell—and are sometimes called ‘bucky-diamonds’ [125].

Following the various cleaning processes, the surfaces of DND particles are usually terminated with oxygen-containing groups, which makes them hydrophilic and helps their stability in aqueous suspensions (see §1c in the electronic supplementary material). This oxygenated surface can be modified by standard chemical methods, replacing the O-containing functional groups with H (producing a mildly hydrophobic surface), with F (which is superhydrophobic) or with NH_2_ (which permits further chemical functionalization) or with a host of organic molecules [126].

Functionalized DND particles are currently of great scientific interest in their own right for a range of applications, e.g. as carriers for targeted drug delivery [126] and as luminescence biomarkers [127,128]. However, in terms of diamond CVD, their main utility is in seeding. Due to their small size, an aqueous or ethanolic suspension of DND particles can be drop-cast on to a substrate surface; after drying, a near monolayer of seeds is formed with extremely high density (10^12^−10^13^ cm^−2^), which is close to the theoretical maximum [120]. Alternative DND seeding methods are also popular—the substrate can be dipped into the DND suspension (with or without sonication), spin-coated or submerged, and the DND particles are left to settle on to the surface. A DND suspension can also be electrostatically sprayed on to a surface enabling complex 3D microstructures to be conformally coated in a layer of seeds [129]. More details about the application of DND seeding can be found in the electronic supplementary material.

Bias-enhanced nucleation (BEN) was considered rather a niche method for nucleation in 2000. However, in the last few years, BEN has come to prominence due to it being used to make freestanding SCD wafers that are 92 mm in diameter [74]. BEN works by applying a negative bias of approximately −100 V to the substrate plate during MWCVD for a short nucleation step. The bias accelerates positive hydrocarbon ions from the plasma bulk, which then strike the single-crystal substrate with energies around 100 eV sufficient to implant C just beneath the surface (a process called subplantation). As time goes on, the concentration of sub-planted C reaches saturation, and the C atoms precipitate out of the surface as diamond—but crucially the diamond islands that form are aligned with that of the underlying substrate lattice. After a few minutes, this nucleation step is complete, the bias is turned off and conditions revert to those for standard CVD growth. The diamond islands continue to grow and then coalesce with very little mismatch at the boundaries, eventually producing a near SCD film across the whole substrate.

**Figure 9.**
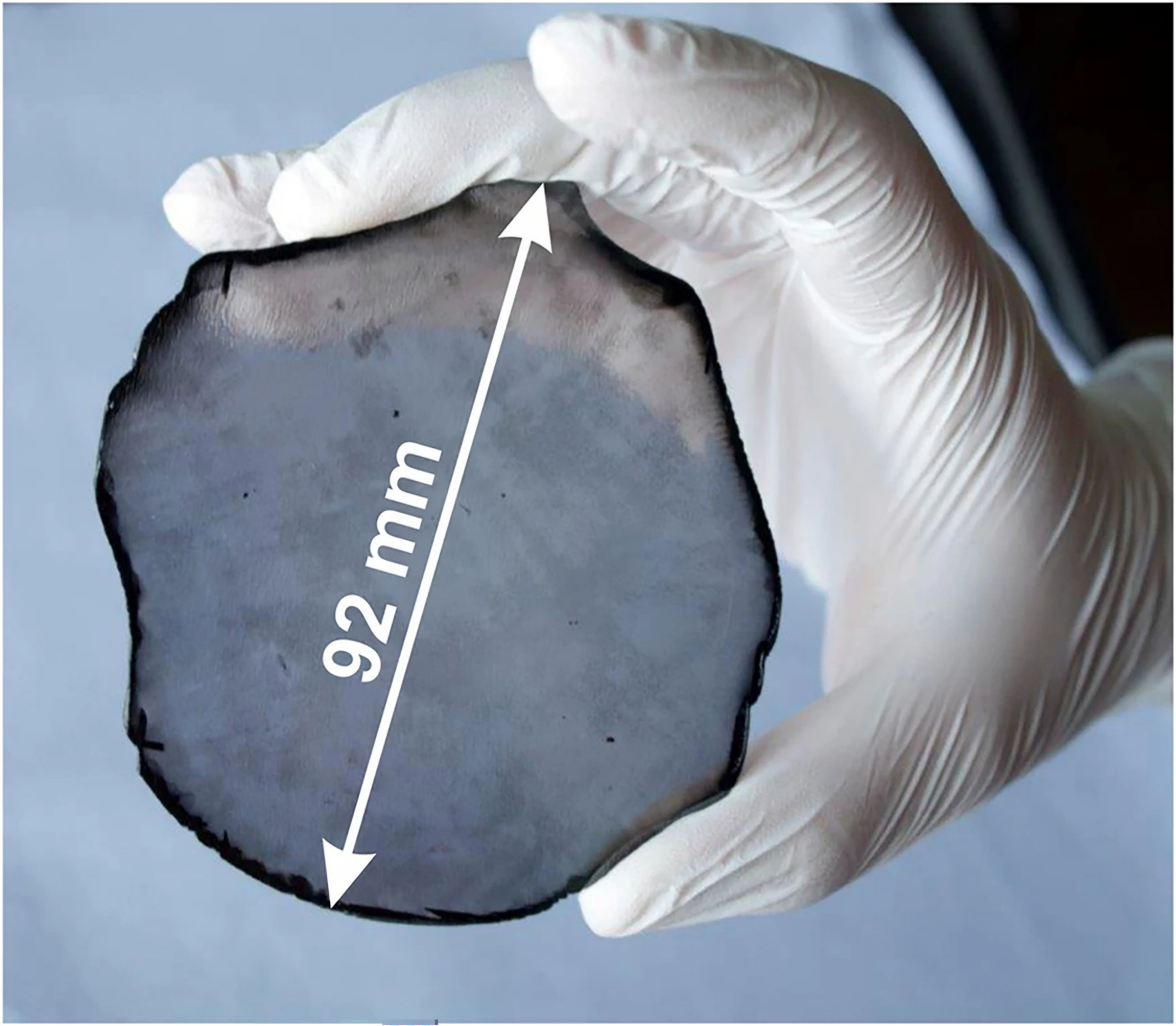
Freestanding unpolished large-area SCD wafer synthesised by heteroepitaxy on Ir/YSZ/Si(001). The thickness of the disc is 1.6 ± 0.25 mm and its weight is 155 carat. Reproduced from [74] under the Creative Commons Attribution 4.0 License.

The original BEN work used Si (100) substrates, and the diamond films produced were called textured diamonds as they were composed of large SCD (100) plates all closely aligned with the substrate lattice [130]. This work was pioneered, among others, by Matthias Schreck from the University of Augsburg, and the big breakthrough was the realization that the nucleation density afforded by BEN was far denser and the crystal orientation distribution much narrower on single-crystal iridium metal substrates. This was partly due to the close lattice match of Ir to diamond [131], but also due to a unique mechanism in which buried lateral growth occurs within an approximately 1 nm-thick carbon matrix induced by the intense ion bombardment, described in detail in [74]. Unfortunately, using freestanding single-crystal Ir substrates is not practicable due to their astronomical cost. However, thin single-crystal Ir layers can be deposited on to various oxides, such as Al_2_O_3_, SrTiO_3_ and MgO [132], with the best being yttria-stabilized zirconia (YSZ). YSZ substrates are available and reasonably inexpensive, but in its bulk form, the CTE mismatch between YSZ and diamond is still rather high. The method of choice, therefore, is to use a triple-sandwich structure by depositing thin layers of YSZ on to a standard cheap Si (001) wafer, followed by a layer of single-crystal Ir, to produce Si/YSZ/Ir substrates. The diamond films grown on these sandwich substrates using BEN followed by MWCVD are near-perfect, transparent single-crystal layers up to 92 mm in diameter by 1.6 mm thick [74] (figure 9).

Large-area freestanding diamond films fabricated by this method are now available commercially from the German company Audiatec [133]. Such heteroepitaxially grown SCD films currently suffer from higher defect densities than their homoepitaxially grown counterparts, as well as significant bowing from the remaining CTE mismatch, but the hope is that SCD wafers with substantially larger diameters and fewer defects can be routinely fabricated by this method in the near future.

## 8. Doping

Undoped CVD diamonds of any grain size are highly electrically insulating. However, doping by incorporation of a suitable impurity atom, usually from Group 3 or 5, can increase the conductivity of the film in a controllable manner. The dopant atoms are usually added to the input gas mixture in gaseous form, e.g. B_2_H_6_ for boron, NH_3_ or N_2_ for nitrogen, SiH_4_ for Si and PH_3_ for phosphorus, or by exposing a solid compound containing the intended dopant (e.g. rods of solid Si or B) to the plasma during deposition. Alternating layers of doped and undoped diamond can be fabricated by switching the doping gas on and off. Dopants in the form of solid rods can be retracted and inserted into the plasma extremely rapidly, allowing very thin (less than 1 nm) dopant layers to be made for so-called ‘delta-doping’ applications (see §12b(ii)).

**Figure 10.**
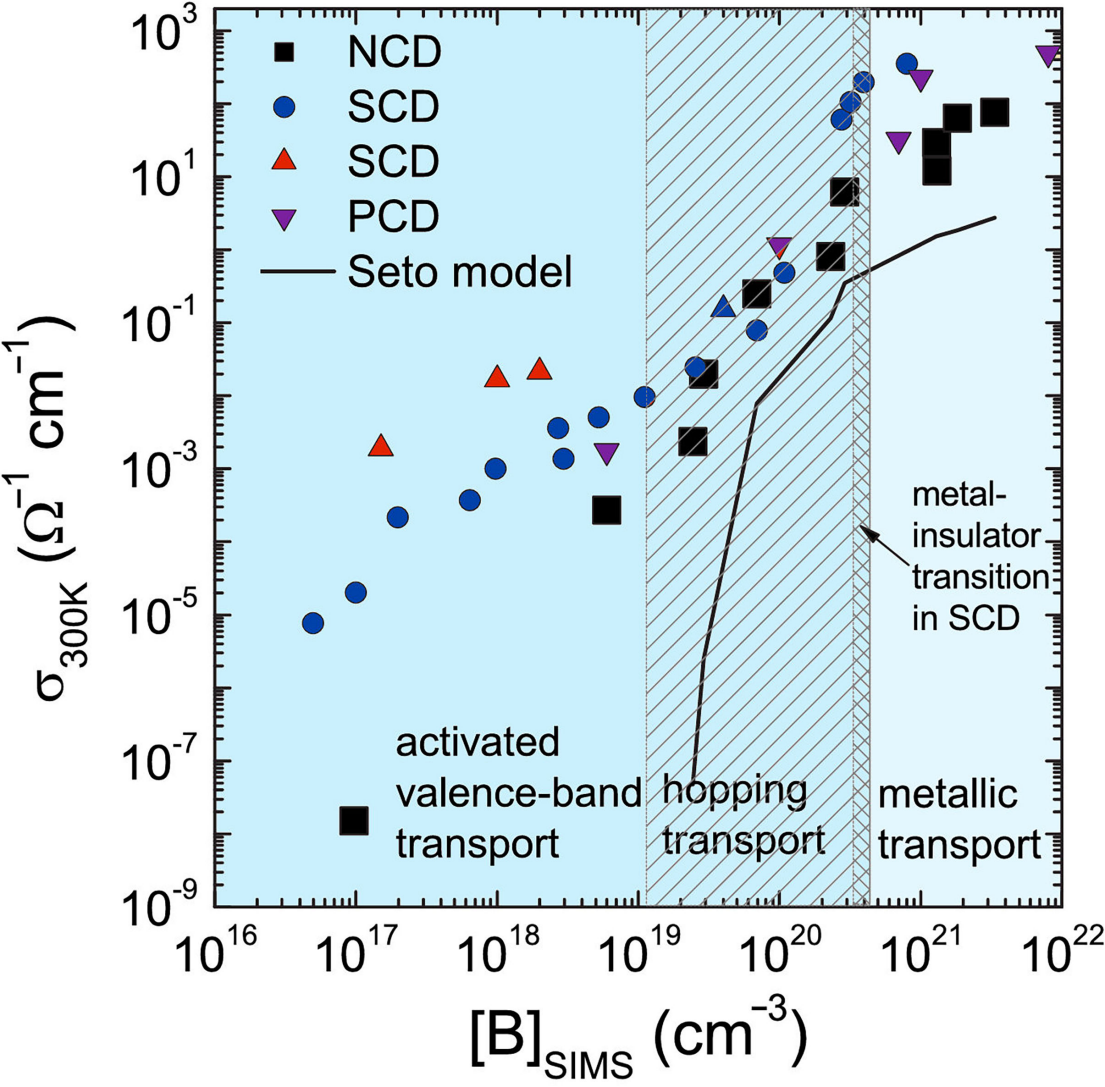
Conductivity (*σ*) at 300 K as a function of boron content measured by secondary ion mass spectrometry (SIMS) for various boron-doped diamond samples. Different conductivity mechanisms operate at different B concentrations, as shown by the labelled regions on the plot. The curve predicted by the Seto model of conductivity is plotted as a solid line. Figure reproduced from [134] with permission and colourised. For [B] > 1 × 10^21^ cm^−3^ and temperatures less than 10 K (not shown) the conduction mechanism switches to superconductivity via Cooper pairs.

Adding boron to diamond changes the conductivity in a reproducible and reliable manner (figure 10) [134]. At low-to-medium concentrations, B doping creates a *p*-type semiconductor [135], while at high concentrations (greater than 1 × 10^20^ cm^−3^), the conductivity becomes near-metallic, and for B concentrations above approximately 3 × 10^21^ cm^−3^, the films become superconducting at temperatures less than 10 K [136,137]. The *p*-type conductivity in B-doped diamond (BDD) results from hole carriers, and SCD exhibits the highest hole mobility at room temperature of any wide-bandgap semiconductor [138]. A higher-mobility material has a higher frequency response because the carriers can travel through the device faster. Also, materials with higher mobility transport more carriers per second, which means they can operate at higher currents and therefore higher powers. Although this high hole mobility in BDD drops considerably with increasing temperature and B concentration, it nevertheless remains high enough for BDD to be used in a variety of simple electronic devices (especially those operating at high frequencies and high powers), sensors and electrochemical electrodes [139].

**Figure 11.**
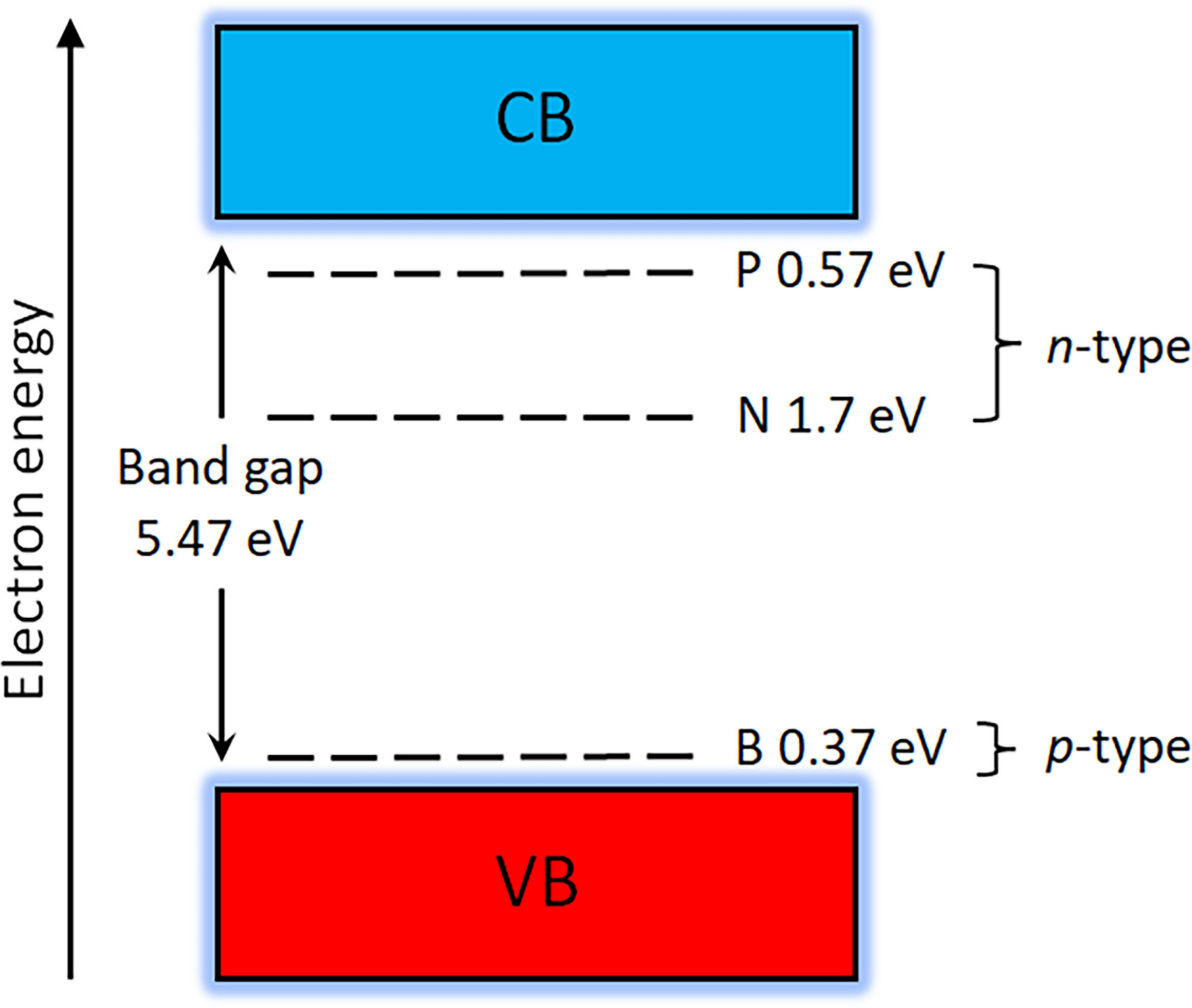
The three most common dopant types (e.g. phosphorus, nitrogen and boron) in diamond with the associated energies within the band gap; VB and CB stand for the valence band and conduction band, respectively. The B acceptor level is measured from the top of the VB, while the P and N donor levels are measured from the bottom of the CB.

In contrast, *n*-type semiconductivity—with electrons now acting as carriers—is much harder to achieve, because most potential *n*-type dopants (P, As and Sb) are too large to easily substitute for a small C atom in the rigid diamond lattice and thus have a low solid-state solubility in diamond [140]. Despite diamond exhibiting high values for electron mobilities similar to the hole mobilities seen in BDD, the low dopant concentration leads to low electrical conductivity and limited device performance. The exception is nitrogen for which atoms are small enough to substitute for carbon [50], but unfortunately, the energy levels of the N donor in diamond are too deep [141] (figure 11) and so are electrically useless for most room-temperature applications. Over the past 20 years, some degree of success has been achieved with phosphorus doping [142], but device performance is still poor compared to competing semiconductor materials such as Si, GaAs, GaN and SiC. Experiments with more unusual candidate dopants, such as Sb [143], Li [144], Na [145], S [146,147] and Se [148], have *all* proven disappointing.

Co-doping—addition of both *p*-type and *n*-type dopants simultaneously—has been attempted—again with little success. B + Li doping failed due to the Li migrating through the diamond (especially when grain boundaries were present), possibly forming Li clusters [149]. Calculations for N + Li co-doping suggested that a LiN_4_ defect, with substitutional Li, is *likely* to behave as a shallow *n*-type donor [150]—if it could somehow be realized experimentally. Similarly, computational predictions for B + N [151], B + P [152] and Se + B [148] look promising, but none have yet been proven experimentally to be useful *n*-type doping schemes.

Despite all this effort, the continued lack of a reliable *n*-type dopant with useful electronic properties at room temperature has hindered the use of diamonds in electronic applications more than any other issue. To date, n-doping unfortunately remains the ‘holy grail’ of diamond semiconductor research.

Another type of conductivity can occur in diamonds, as a result of an unusual property of the diamond surface, called ‘surface transfer doping’ [153,154]. Hydrogen-terminated diamond exhibits a surface dipole due to the difference in electronegativity between the carbon atoms in the bulk and the H atoms on the surface. Electron-accepting molecules from the ambient air adsorb on to this polar surface, and electrons are transferred from the bulk diamond to the adsorbates. As a result, the adsorbates become negatively charged. Similarly, the diamond becomes positively charged, and this takes the form of a stable two-dimensional hole-gas (2DHG) layer a few nanometres thick, which forms just below the surface [155]. This layer is electrically conducting, with both a high hole mobility (100−200 cm^2^ V^−1^ s^−1^) and a high hole concentration (10^12^−10^13^ cm^−2^). Unfortunately, the conductivity can be altered or even destroyed by simply changing the atmosphere (pressure, humidity, etc.) above the surface, as this affects the concentration and nature of the adsorbates. To stabilize this fragile conductive layer, the diamond surface can be capped with a protective layer of an electron-accepting oxide, such as V_2_O_3_, Al_2_O_3_ or MoO_3_ [156]. This capping layer hermetically seals the surface as well as providing additional surface acceptors helping to generate and stabilize the 2DHG layer. Several groups have recently exploited this process to fabricate novel types of electronic devices (see §12b(ii)).

## 9. The NV centre

**Figure 12.**
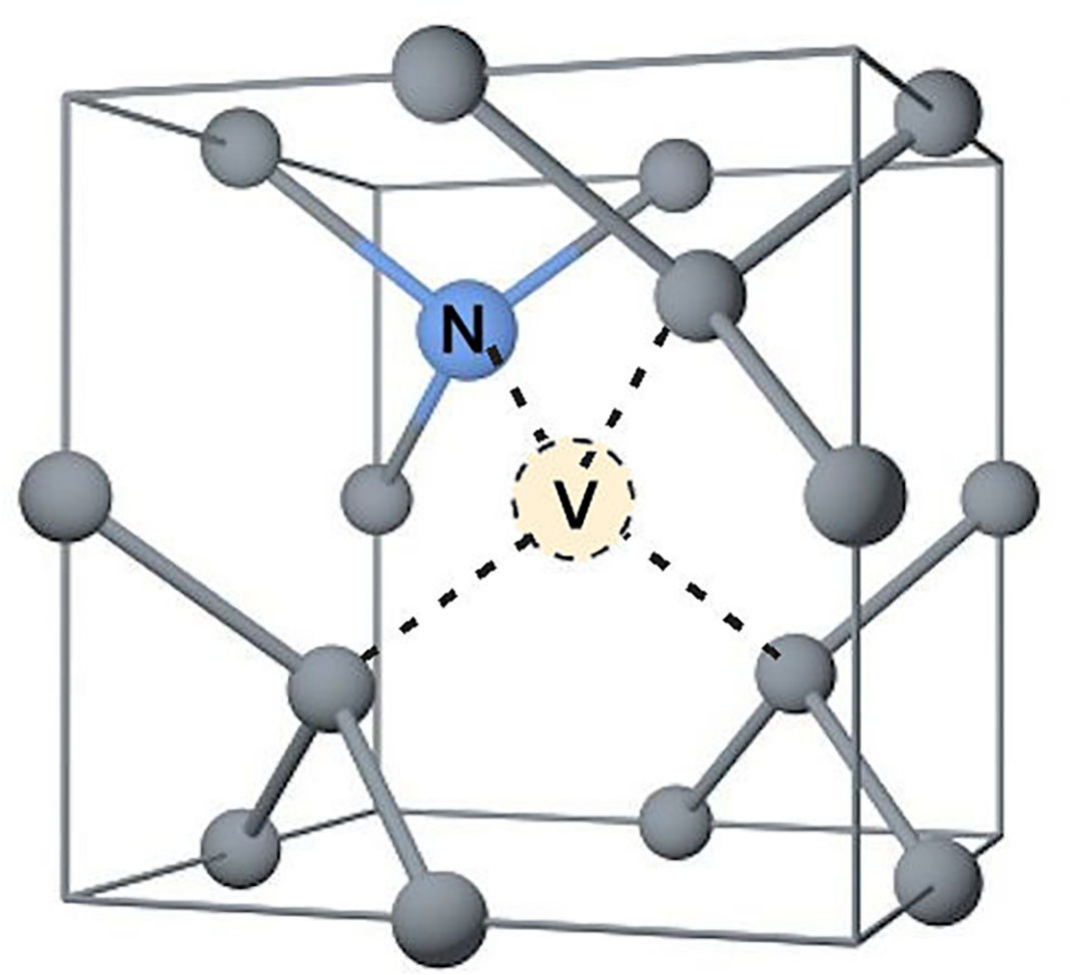
Schematic diagram of the NV defect centre in the diamond unit cell.

Although its usefulness as a dopant is doubtful, N in diamond, when situated next to a vacancy, forms the so-called ‘NV centre’ [157], as shown in figure 12. The negatively charged version of this defect (NV^−^) is causing a great deal of excitement in the scientific community because these defects behave as isolated ‘pseudo-atoms’, with a set of energy levels distinct from those of the surrounding diamond. NV centres act as excellent single-photon sources [158], underpinning a host of novel applications involving quantum computing and quantum information processing [159,160] (§12c), as well as quantum sensing and magnetometry [161] (§12c(iii)). When excited by green laser light, the NV centres fluoresce in red, and this gives rise to another set of applications for the study of biological cells [162] (§12c(iii)).

There are a variety of methods to fabricate NV^−^ centres in diamond. The most popular is to implant diamond with N ions, followed by high-temperature annealing. The implantation process adds N to the diamond, with many of the N atoms displacing C atoms from the lattice and taking their place as substitutional atoms. The implantation process also creates damage, such as vacancies and interstitial C atoms. The subsequent anneal then allows the defects to relax into a stable structure, and because the formation energy of an NV^−^ centre is exothermic, these form readily. The difficulty is in not flooding the diamond with too many N atoms such that the density of N atoms is so great they start to cluster. As such, simultaneous implantation of only a few N atoms (less than 5) within a volume of approximately 1 μm^3^ is required—which is pushing the limits of current implantation technology. Furthermore, the NV centres need to be within a few nanometres of the surface for them to be affected by the presence of any adsorbates (e.g. for sensing purposes) and for any emitted photons to be able to escape the surface [163]. The NVs also need to be separated from any neighbouring NVs by more than 30 nm to prevent dephasing (signal interference between the two centres) [164]. The substrate must be ‘electronic grade’ or ‘quantum grade’—extremely high-quality SCD, which is transparent at the wavelength of the emitted photon with no optical defects (such as birefringence), contain extremely low N concentrations ( less than 1 ppb) and polished to a smoothness of a few nanometres or better. Along with gemstones, these exacting requirements for electronic-grade diamonds for quantum applications remain one of the key driving forces for improvements in diamond growth technology.

As well as the NV^−^ centre, several other diamond ‘colour centres’ have been investigated for quantum applications, including NV^0^ (neutral NV) [165], SiV^−^ [166], SiV^0^ [167], GeV^−^ [168], SnV^−^ [169] and PbV^−^ [170], and many more candidate defects are being discovered on a regular basis [171].

## 10. SCD growth

One of the most remarkable success stories in diamond technology over the past 20 years is the development of methods to grow SCD gemstones of sufficient size and clarity so that they can compete directly with natural diamonds in the jewellery market. The market for CVD lab-grown diamonds was valued at $11.3 billion (USD) in 2022 and is projected to reach $15.9 billion by 2027, growing at an extraordinary rate of approximately 7% a year [172]. This burgeoning demand for SCD gemstones has been the driving force behind significant improvements to both the growth technology (see §3) and the quality and size of high purity diamonds available as substrates for scientific use (see §11). The three crucial considerations for both the gemstone market and scientific applications of SCD are high growth rate, high throughput (either large surface area or more diamond gemstones per growth run) and high purity.

### (a) High growth rate and throughput

For gemstones, SCDs up to 1 cm in thickness can now be deposited by MWCVD at growth rates up to 100 μm h^−1^ [173], although more typical routine industrial growth rates are approximately 20 μm h^−1^. For SCD growth, the growth chemistry is modified only slightly from that used for polycrystalline films. CH_4_/H_2_ is still the preferred gas mixture, except that for high growth rates the carbon concentration in the gas phase is often increased from 1% to approximately 15% CH_4_ in H_2_, while process pressures are also increased to over 200 torr. To prevent build-up of longer-chained carbon radicals in the gas phase, which might cause unwanted renucleation, the plasma density is increased to typically 70−300 W cm^−3^ by using higher-power MW systems. Such high-power (up to 75 kW) 915 MHz reactors can accommodate large substrate platens capable of holding as many as 24 diamond seeds at a time, substantially increasing throughput. Often, small amounts of O_2_ are added to the gas chemistry to further improve the diamond quality, while additions of small quantities of N_2_ improve growth rates significantly (see §4b), leading to diamond with a yellowish or light brown colour. These unwanted colours can be partially or wholly removed using a post-growth treatment to produce a clear colourless gemstone [174]. Indeed, most CVD material sold as jewellery has undergone at least one round of post-growth treatment, such as HPHT annealing or a combination of irradiation and annealing.

**Figure 13.**
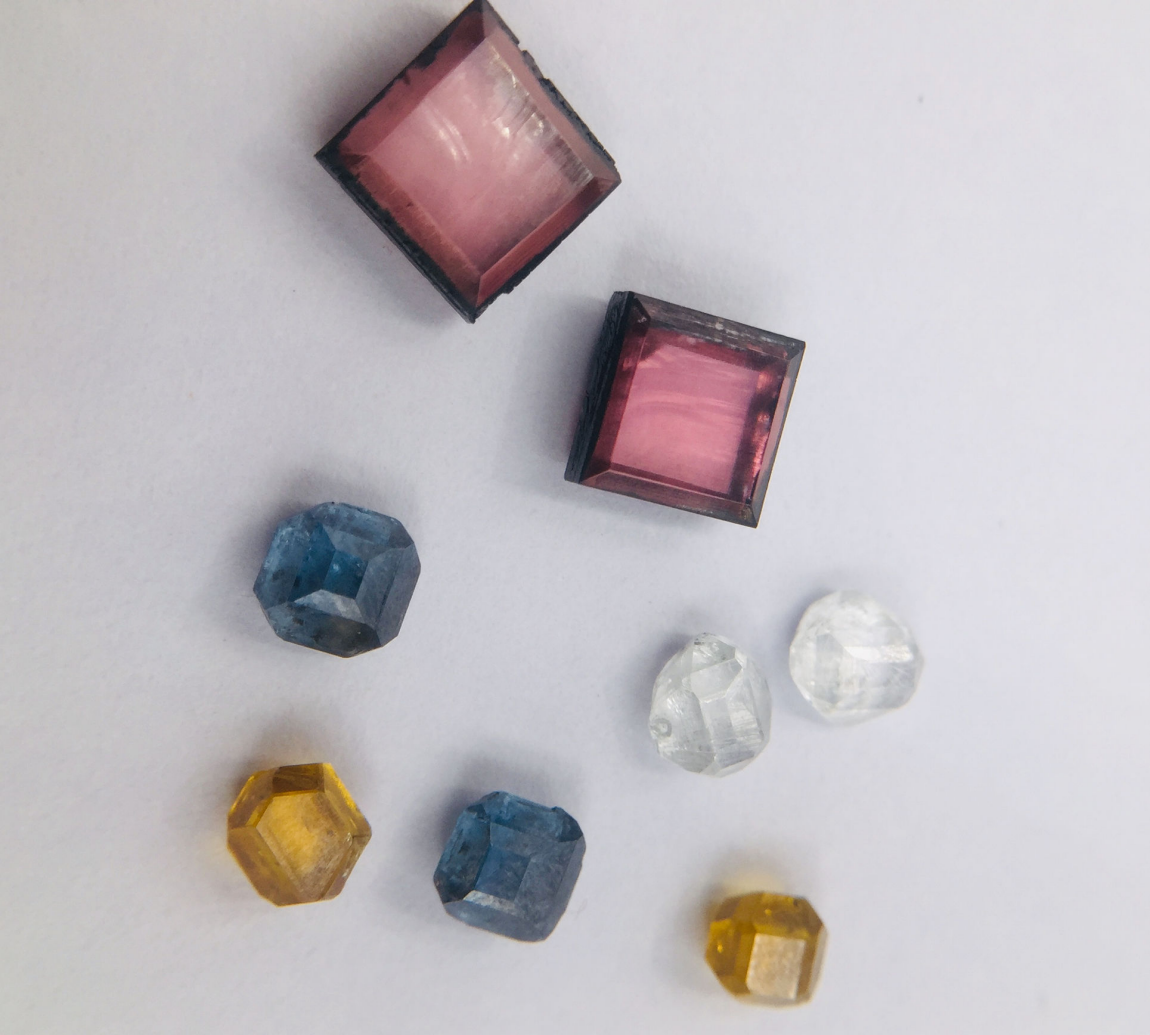
Examples of fancy-coloured CVD diamond gemstones. Photo reproduced with permission of Infi Advanced Materials Co., Ltd [176].

After decades of studying colour centres in natural diamonds, it is now possible to engineer the correct impurities into CVD diamonds during growth or in post-growth processing, to produce diamond gemstones with specific colours [175]. These so-called ‘fancy’ diamonds can sell for many times the price of their colourless counterparts, and so some manufacturers concentrate on producing these diamonds exclusively. Fancy diamond colours include brown (attributed to non-diamond carbon inclusions and/or internal extended defects such as dislocations), yellow (due to incorporation of nitrogen), red (from post-growth irradiation), blue (due to boron incorporation) and grey (boron again, plus sometimes carbonaceous inclusions; figure 13) [176]. The most famous natural blue diamond is, of course, the 46-carat Hope Diamond, which is permanently on display in the Smithsonian Museum in Washington DC [177]. If the rapid rate of progress in CVD gemstone growth technology continues, a very large, blue, lab-grown diamond should be possible within a few more years [178] (hence a possible secondary meaning for the title of this paper—a new Hope?).

### (b) High purity

This is achieved in various ways. Ensuring that high-quality vacuum components are used throughout the MWCVD process prevents air leaks. The use of ultra-high purity process gases (99.9999%) and hydrogen generators—which generate extremely clean H_2_ gas (99.99999%, i.e. an impurity content less than 100 ppb) by electrochemically splitting high-purity water—ensure that unwanted impurities in the diamond, especially nitrogen, can be reduced to levels below 0.1 ppb [24]. Extremely stable noise-free MW power supplies and control electronics ensure that growth conditions (pressure, gas flows, power and temperature) remain constant to within fractions of a per cent during the many hours or days required to produce thick films or large gemstones.

However, extended defects, such as dislocations, grain boundaries, twins and cracks, remain a significant problem in SCD growth [179], as these can substantially reduce the diamond quality and cause a variety of problems, such as birefringence [180], unwanted luminescence or carrier leakage in diodes [181]. SCD is usually deposited epitaxially on to mechanical-grade HPHT type-Ib seeds due to their low-cost and availability. Unfortunately, these substrates contain large numbers of point defects (impurities such as N and metal atoms) and extended defects, especially dislocations, that significantly affect the CVD films grown upon them. The dislocations in the substrate are a problem because they transfer to the growing CVD film [182] and ‘thread’ through the layer along the (001) growth direction.

The dislocations at an HPHT seed surface can be revealed and their density determined by a short dry-etch process using an H_2_/O_2_ plasma. This process preferentially etches the dislocations projecting from the surface and creates micron-sized inverted-pyramidal square-sided etch-pits visible via optical microscopy [183]. Typical dislocation densities in HPHT seeds are 10^2^−10^4^ cm^−2^, increasing to 10^5^−10^7^ cm^−2^ for CVD homoepitaxial SCD, and further to 10^8^−10^10^ cm^−2^ for heteroepitaxial SCD [182,184]. However, not all defects have the same detrimental effect. Defects that fatally disrupt the operation of the application in question are termed ‘killer’ defects [185]. The exact nature of what constitutes a killer defect differs depending upon whether the application is optical or electronic, as well as between different types of electronic devices. The typical density of killer defects is 10^4^−10^5^ cm^−2^ for Schottky junction devices fabricated on a Ib type HPHT substrate [186], i.e. killer defects constitute approximately 1–10% of all dislocations in the sample. For comparison, the lowest killer defect density reported in a device to date is approximately 600 cm^−2^ [187]. For a particular application, identifying which of the various defects present in the diamond are the killer defects that must be eliminated (or at least minimized) and which are less harmful is currently a major endeavour if SCD devices are to live up to their promise [188].

There are two methods commonly used to significantly reduce the generation of dislocations at the interface between the seed/substrate and the CVD diamond layer. The first involves polishing or etching the seed/substrate prior to deposition to reduce the number and severity of surface defects [189]. The second method involves selecting much higher-quality type-IIa HPHT diamonds with low dislocation density for use as seeds [190]. Other reported methods, which have also been used for heteroepitaxial CVD on Si/YSZ/Ir substrates, include blocking the threading dislocations using thin Au stripes [191], deliberate incorporation of W impurities [192] or a grid of laser-cut holes [193] or Ni etching [182] followed by lateral overgrowth of more diamond. Sometimes the easiest solution is to simply grow the diamond film thick enough that most of the dislocations grow out and disappear [184].

With suitable seed selection combined with substrate pre-treatment and the use of clever growth technology, reports are starting to emerge of CVD SCD films with very low dislocation density (400 cm^−2^) [194]. Despite such impressive improvements in defect reduction, current state-of-the-art values are still a long way from those required for realistic commercial SCD devices [188], and this will become one of the major challenges for diamond device fabrication in the coming years.

Since about 2008, CVD diamonds of gemstone quality began to enter the marketplace, but it was not until around 2013 that production really took off, particularly in China, which now manufactures approximately 50% of the lab-grown diamonds used in jewellery worldwide [11]. Gem manufacturers in other countries (see electronic supplementary material, table S1), such as India, Singapore, the UK and the USA, are also ramping up their output of diamond gemstones. The majority of faceted CVD gemstones on the market today are 2 carats or less in size, although the average sizes are increasing each year [175]. The current record for the largest facetted CVD lab-grown gemstone diamond is held by a stone called ‘The Pride of India’ produced by Ethereal Green Diamond (Mumbai) in May 2023; it weighed 34.59 carats and measured 13.97 × 13.87 × 9.56 mm [195], while the largest equivalent HPHT diamond gemstone weighs slightly less at 20.23 carats and was made in 2019.

Lab-grown CVD or HPHT diamonds can be distinguished from natural diamonds by a number of identifying markers caused by their growth conditions [196,197]. Some differences, such as metal inclusions in natural stones, can be seen in an optical microscope or become apparent from the interference patterns visible using polarised light microscopy. CVD diamonds also have a different fluorescence signature to natural diamonds and nowadays can be readily detected using portable systems such as the *DiamondView* imaging instrument developed by de Beers in the mid-1990s [198,199]. More details about methods of distinguishing natural and CVD diamonds can be found in §2 of the electronic supplementary material.

## 11. Large-area diamond films

To compete against other materials and for the commercial advantages in price, reproducibility and throughput that accompany larger area growth, it is essential that diamond films are fabricated in the form of large wafers and to thicknesses approaching 1 mm. The Si industry currently uses 12-inch single-crystal wafers as standard, while materials such as SiC and GaN are not far behind and have established commercial products with approximately 6-inch wafers. It seems that SCD substrates—which appear to have been stuck at only 10 × 10 mm for many years—have a long way to catch up. Fortunately, there are a number of strategies proposed for large-area diamond growth described below and reviewed in detail in [4].

### (a) Polycrystalline diamond films

Current MWCVD reactors are limited to substrate areas approximately 4 inches in diameter, although larger areas are possible using lower frequency MW power supplies (see §3). However, the requirement for a tuned cavity in MW systems makes large-area growth problematic. China is currently leading the way in DC arc-jet technology for large-area polycrystalline diamond deposition [11]. In a new type of arc-jet reactor (or plasma torch) developed by the University of Science & Technology Beijing, large-area uniformity is achieved through an externally applied magnetic field that stabilizes the arc jet, combined with careful magnetic and fluid dynamic control to guarantee the dynamic mixing of gases before they strike the substrate [12]. The 100 kW models are available that are capable of depositing diamond films over a substrate of 150 mm diameter with a thickness of 3 mm at growth rates as high as 40 μm h^−1^. The high rate of gas usage in these types of reactors is mitigated by recycling 95% of the process gases. Such reactors are currently employed in China for the mass production of mechanical, thermal and optical grade (but not electronic grade) diamond film wafers [11]. Plans to scale up to even larger areas are undoubtedly in the pipeline; however, the enormous power supplies that would be required, along with the vast cooling rates and gas flows, may prove difficult hurdles to overcome.

Commercial hot-filament CVD reactors are available that can deposit polycrystalline diamond films on to 12-inch-diameter wafers; however, their low growth rates (a few μm h^−1^) mean they are not practicable for mass-production applications. The various distributed antenna reactors (LAPD, DAAM and SPW) described in §3 have the potential to grow on very large areas, perhaps several m^2^. However, the diamond quality is poor (NCD at best), and the growth rates are pitifully low. Nevertheless, such systems may find use in coating large-area panes of glass with a thin protective NCD layer.

### (b) Heteroepitaxial SCD

The most promising candidates for SCD films grown on non-diamond substrates to date are those grown upon Si/YSZ/Ir substrates using BEN and MWCVD [74] (see §7). Transparent freestanding SCD diamond films have been grown with diameters as large as 92 mm (figure 9); however, larger areas are proving tricky due to challenges with cracking and bowing resulting from CTE mismatch between the diamond and substrate. The large number of defects that propagate into the diamond from the imperfect interface between the diamond and the Ir is also a problem for electronic or quantum applications. Although improvements in film quality and size are reported regularly, it remains to be seen if this fledgling technology can compete with the SCD films grown homoepitaxially.

### (c) Homoepitaxial SCD

The problem with growing large-area SCD on diamond substrates is that the seeds are usually made by the HPHT method and are thus limited in size to a maximum of approximately 12 × 12 mm [200]. Even if larger seeds could be fabricated, the other issue is that the substrate size remains limited by the size of the MW chambers (see §3d). For smaller substrates (approx. 1 cm^2^), there are a number of options for faster growth rates. Diamond CVD growth rates generally scale with power input [201], so simply increasing the power density of the MW plasma can greatly increase growth rates, although care has to be taken with cooling to ensure the growth temperature remains within the usual limits. Similarly, in HFCVD, increasing the filament temperatures to nearly the melting point of the Ta filament (approx. 3000 K) achieved growth rates of 10 μm h^−1^, but at the expense of some evaporated metal from the filament contaminating the diamond [202]. Adding a small amount of nitrogen-containing gases to the feedstock can more than double the growth rate and produce flat (100) diamond surfaces (see §4b). Another approach is to grow via the step-flow growth mode on miscut substrates [203]. These substrates have been deliberately cut off-axis by a few degrees such that there are many step-edges present on the growth surface. CVD then proceeds via the step-flow mechanism at these edges, which speeds up the growth rate and reduces hillocks [204], permitting very thick, macroscopically smooth films to be deposited [205].

**Figure 14.**
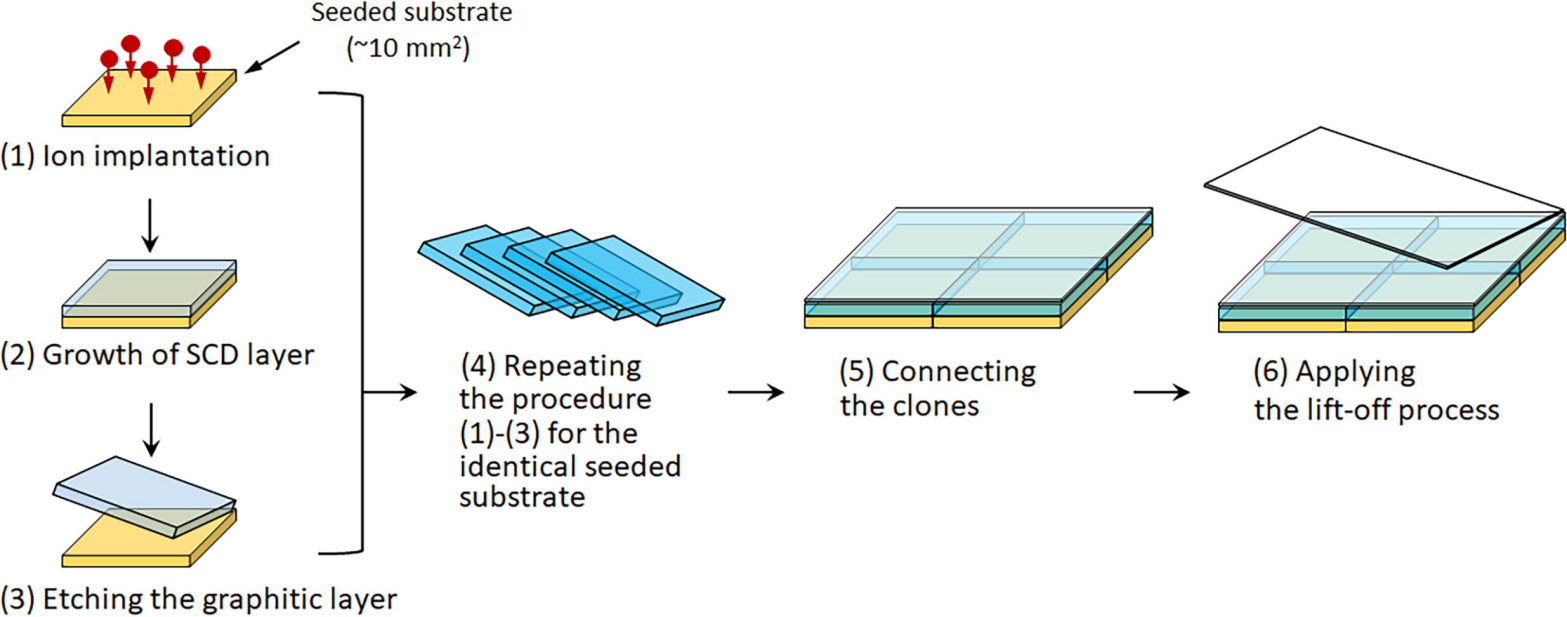
Procedure to produce clones and tiled clones. Figure redrawn based upon diagram in [206].

The ability to rapidly grow large numbers of thick plates of 10 × 10 mm^2^ SCD in high-power MW systems promises perhaps the most hopeful method for fabricating large-area SCD substrates—‘mosaic growth’. In this process, many diamond seed plates are arranged in a grid in the reactor, and diamond is grown laterally, fusing them together into one large SCD plate. The resultant wafer is formed of small tiles, but the plates need to be aligned accurately with each other and have identical off-axis orientation. One method to achieve this is the use of 'cloned' tiles (figure 14). This technique uses a lift-off method first reported by Parikh *et al*. [207], in which a high-energy ion beam implants C ions into a flat SCD seed forming a thin, damaged layer buried beneath the surface. Annealing in vacuum at approximately 600°C graphitises this layer, making it much more amenable to etching than the surrounding diamond. This graphitic layer can then be etched electrochemically, lifting off the freestanding SCD plate above it. Another CVD growth run then creates a new homoepitaxial layer, which can then be implanted and removed again. This process allows for repeated growth of SCD clone plates that have identical characteristics. These can then be aligned and grown together into a single, large tiled clone [206] (figure 14). Tiled clones still contain imperfect crystal boundaries, so the next step is to use them as an even-larger-area seed crystal for another subsequent homoepitaxial growth. To remove the newly grown larger area layer from the top of the tiled mosaic, the surface is mechanically polished then ion implanted as before, followed by a final electrochemical lift-off process. Although this is a complex multi-step process, SCD wafers of size 60 × 40 mm^2^ have been produced this way, which consist of 24 SCD plates, each 10 × 10 mm [206]. A number of companies (e.g. EDP Corporation in Japan) now sell mosaic diamond substrates with areas approximately 25 × 25 mm^2^, and it seems to be only a matter of time before even larger areas become available.

## 12. Applications

One of the most remarkable things that has occurred in CVD diamond technology over the past 20 years is the emergence of an astonishing variety of commercial applications for this material. Diamond has always been seen as ‘the biggest and the best’ with regard to most physical and mechanical properties [1], and these extreme properties are now beginning to be exploited in a wide range of exciting new uses. Indeed, the number and variety of applications for diamonds is now so large that they require an entire review article all to themselves [5]. Thus, here we will focus only on the three applications that we believe show the most promise to produce transformative widescale technologies: quantum information, electrochemistry and electronic devices.

### (a) Electrochemistry

**Figure 15.**
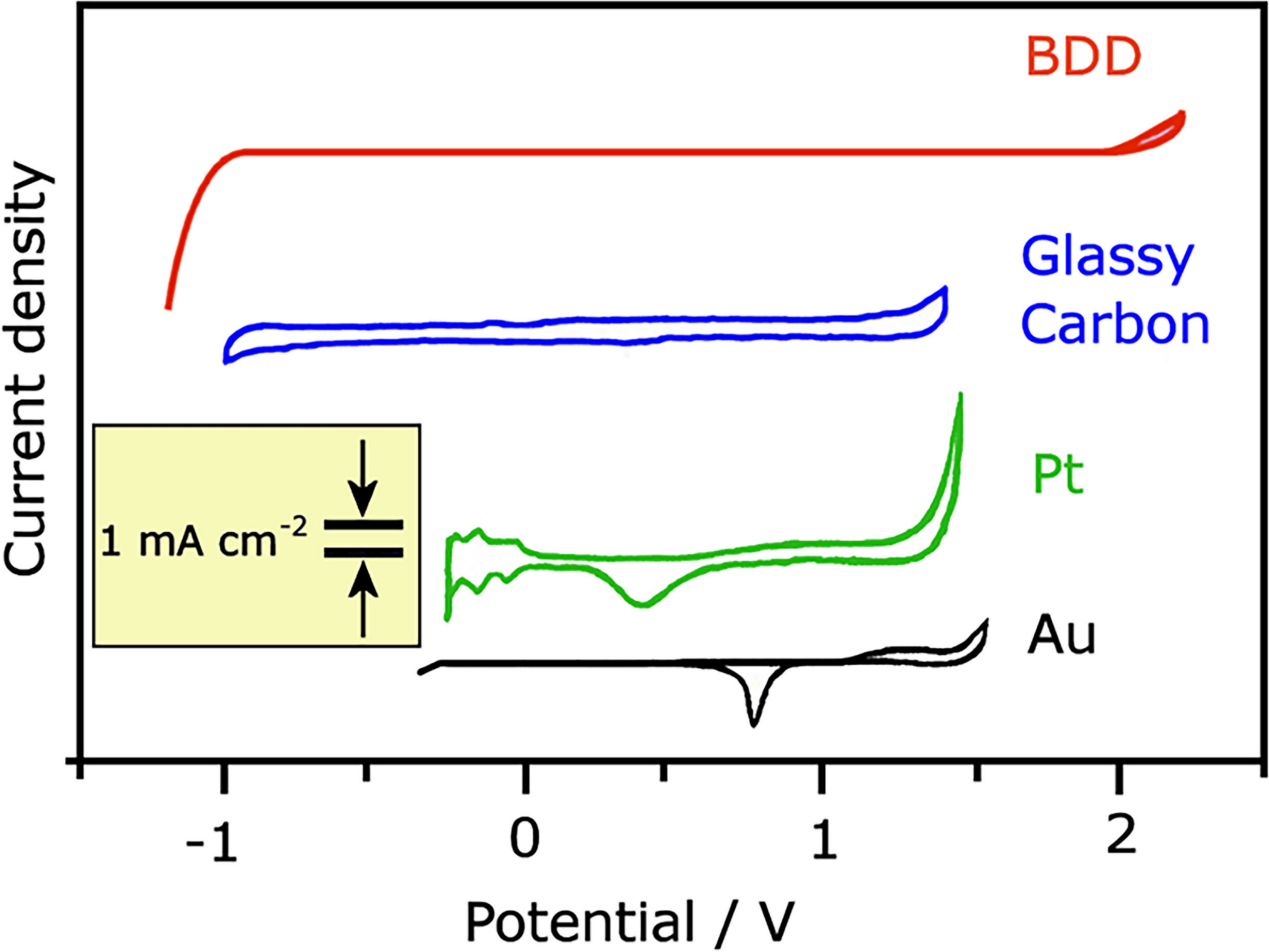
The potential windows of several different electrochemical electrodes. Redrawn using data from [210].

**Figure 16.**
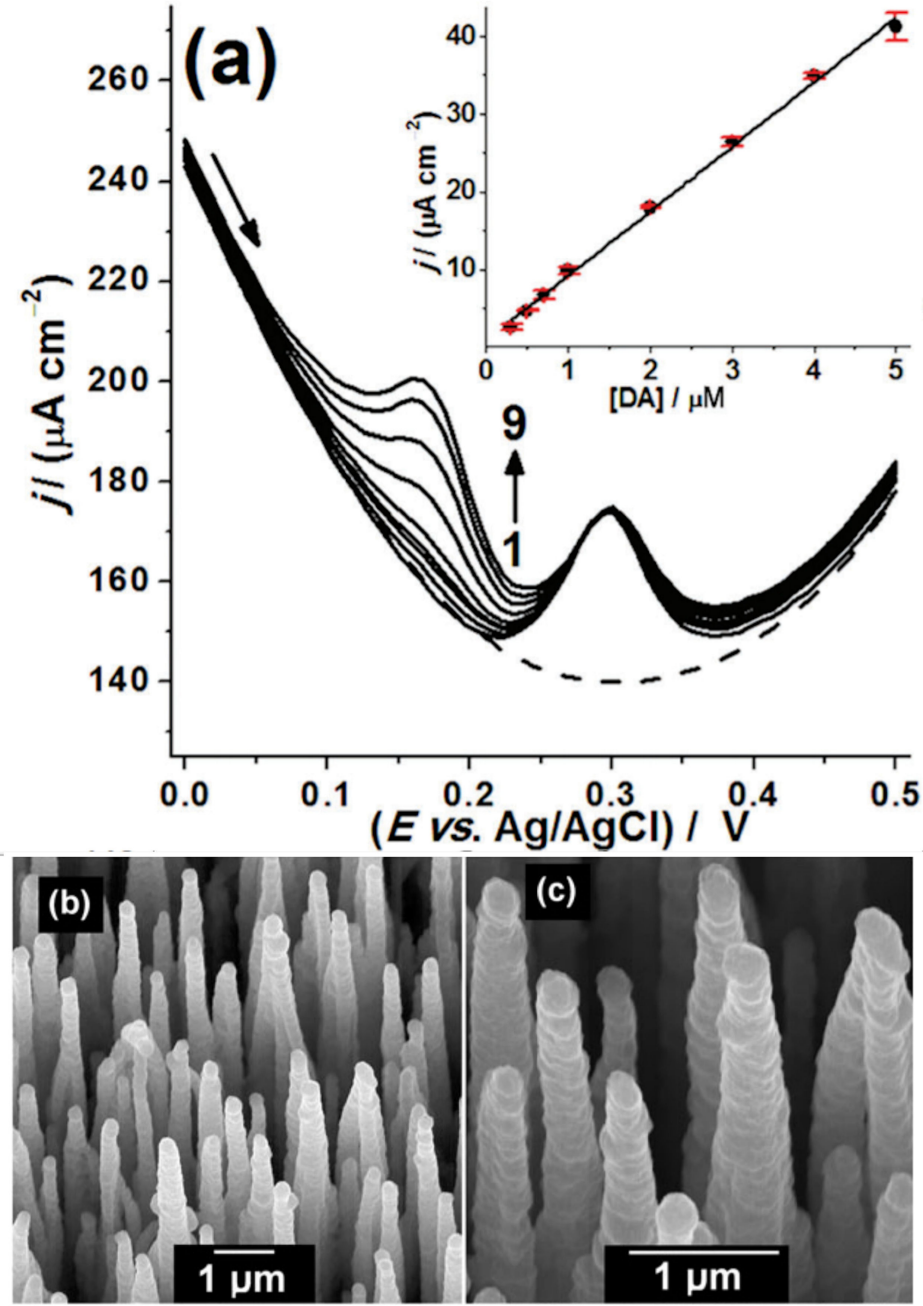
An example of the sensitivity and selectivity of BDD electrodes used for electrochemical trace analysis in water. (a) Differential pulse voltammograms recorded for different concentrations of dopamine (DA) (1–9 = 0.0–5.0 × 10^−6^ M) in the presence of a chemically similar analyte (3.0 × 10^−5^ M uric acid). Inset: current density *j* (mA cm^−2^) versus concentration of dopamine (mM) showing a linear response with concentration. (b,c) SEM images of ‘black diamond’, nanostructured BDD needles on the surface of an electrode, with electrochemically active area 220 times larger than that of a flat diamond electrode. Figures reprinted under CC BY 4.0 licence from [211]. (Published by The Royal Society of Chemistry).

Electrodes made from or coated with BDD have a number of advantages for electrochemistry compared to conventional electrodes made from gold, platinum or glassy carbon [139,208,209]. First, BDD electrodes have a wide potential window from −1 to +1.8 V (figure 15), allowing detection of redox species that would normally fall outside the operating range of conventional electrodes [210]. Within this operating window, the response is flat, so there is no background and little to no capacitance, making BDD electrodes highly sensitive. This enables them to detect the presence of compounds at nanomolar (ppb) levels, even in the presence of chemically similar species (figure 16a). Also, BDD electrodes are chemically inert and non-corroding at high temperatures, high pressures and in environments where other electrodes would not survive. These include biological environments in which diamond is bioinert, i.e. its presence does not invoke an unwanted biological response, such as inflammation. Furthermore, BDD electrodes can be nanostructured into various shapes, such as nanowires [212], foams [213] fibres [214] and needles [211], increasing the available surface area by up to 1000-fold, along with an accompanying increase in sensitivity [211] (figure 16b,c). In addition, BDD electrodes are robust, have far less tendency to foul than other electrodes and can be electrochemically cleaned *in situ*.

However, it should be noted that BDD comes in many different varieties, and the electrochemical performance of the electrode depends critically upon grain size, B-doping level, surface functionality (e.g. hydrophilic H-termination or hydrophobic O-termination), surface roughness, number of grain boundaries and their *sp*^2^ carbon content, among other factors [139]. For some applications, particularly those involving high electrical currents such as water purification (§12a(vi)), delamination of the BDD film can be a problem.

There are perhaps 50 electrochemistry research groups worldwide using BDD electrodes, and a number of companies (e.g. Element Six (UK), Windsor Scientific (UK), Adamant Technologies (Switzerland), Condias (Germany), Sumitomo (Japan) and sp3 Diamond Technologies (USA)) sell BDD electrode material for use as electrodes.

#### (i) Electrochemical analysis

One of the most important uses for BDD electrodes is to determine the identity and concentration of unwanted impurities in a solvent, especially those in water supplies such as rivers and lakes. Many toxic compounds and metals have redox potentials that are too high to detect using conventional electrodes because the oxidation and/or reduction of water occurs at lower potentials than those required to detect the target compound. BDD electrodes, with their wider potential window, have been used to analyse a range of aqueous systems, including detection of heavy metals (mercury [215], cadmium, lead, nickel [216] and arsenic [217]), polycyclic aromatics and pesticides [218], hormones and estrogenic compounds, explosives [219], neurotransmitters such as dopamine [211], drugs (paracetamol, narcotics and pharmaceuticals) [220] and water-soluble nerve agents [219].

#### (ii) *In situ* medical diagnostics

An emerging application for BDD electrodes, especially in neuroscience, is for electrochemical measurements made *inside* a living organism. Micron-sized electrodes are required [221], prepared by coating a sharpened metal W or Pt wire with BDD. Such *in situ* methods provide important information about the changes in the concentration of electroactive neuro-signalling molecules near the site(s) of their release and/or action. BDD microelectrodes have been used to measure the release of norepinephrine from sympathetic nerves (a process that regulates the diameter of arteries and veins) [222], as well as serotonin release from enterochromaffin cells in the small and large intestine [223]. One issue with using BDD for *in vivo* sensing is the rigid structure of diamond that is undesirable for sensors implanted into soft tissue. To overcome this, Fan *et al*. [224] have created a flexible BDD microelectrode using parylene C as a soft substrate. This flexible sensor is capable of electrochemical detection of dopamine in the presence of ascorbic acid and has been used to record the *in vivo* neural activity of a rat. Although such studies are still in their infancy, they hold great promise for future medical diagnostics.

#### (iii) Modified B-doped diamond electrodes

Although diamond electrochemistry using planar macroscopic diamond films has been widely investigated, the use of diamond nanostructures such as nanotextures, nanowires, networks, porous films, nanoelectrodes and undoped/BDD nanoparticles is also a thriving field of study (reviewed in detail in [225]). Similarly, BDD electrodes decorated with metal nanoparticles are another important field, as the metal nanoparticles can be utilized to study specific analytes while the BDD electrode acts as an electroactive but chemically inert support. There are many examples of the use of nanoparticle-decorated BDD electrodes for a variety of analytical applications (reviewed in [208,226]), but some recent representative examples include the use of Pt (biosensors [227]), Ag (analysis of ceftizoxime [228]), Au (proton detection [229], tumour-marker sensing [230], Hg detection [231]), Ni (immunosensor [232]) and Bi (Zn, Cd, Pb ion detection [233]).

#### (iv) CO_2_ reduction

BDD electrodes can also be used to expedite useful electrochemical reactions [208]. To improve efficiency, often the bare BDD electrodes are decorated with metal or metal-oxide nanoparticles to exploit the catalytic activity of the high-surface-area nanoparticles and to make the reaction more selective. Also, the low background current of BDD electrodes suppresses the production of H_2_ gas, which might otherwise compete with the electrochemical reaction of interest. For example, there is a lot of interest currently in capturing CO_2_ from the air and converting it into chemically useful compounds, such as formic acid, formaldehyde or methanol [234]. Besides reactivity (especially at low overpotentials) and stability, the major issue is the catalyst’s ability to selectively produce a single compound with high efficiency. A recent example [235] reported that CeO_2_-decorated BDD electrodes allowed CO_2_ electrochemical reduction at overpotentials below 50 mV, yielding formic acid with a Faradaic yield higher than 40% and with stable performance for many hours. Electrolyte composition and concentration also has an effect upon the efficiency of the CO_2_ reduction reaction, as reported by Jiwanti & Einaga [236], who used Pt-decorated BDD electrodes with NaCl and KCl cathode electrolytes. Ir-decorated BDD electrodes were used to produce formic acid with a Faradaic efficiency of approximately 50% and a low overpotential of −1.7 V [237]. Other C_2_- and C_3_-containing molecules, such as ethanol (main product), acetaldehyde and acetone, were produced from the electrochemical reduction of CO_2_ at the surfaces of Cu-modified BDD electrodes in aqueous media at room temperature, with the product distribution being dependent upon the amount of deposited Cu and the applied potential [238].

#### (v) Electrochemical water splitting

Electrochemical water splitting (EWS) is electrochemically converting water into H_2_ and O_2_ gases. The H_2_ can then be reacted with oxygen in fuel cells to produce electrical power. Thus, development of a cheap and efficient EWS process is key to hydrogen power generation and the ‘hydrogen economy’ [239]. Unfortunately, the wide potential window of BDD electrodes, which is so useful in electroanalysis, is detrimental to EWS, as it means a high overvoltage is required for water splitting [240]. The two key processes involved in EWS—the hydrogen-evolution reaction (HER) and the oxygen-evolution reaction (OER)—are both inner-sphere reactions that consist of multi-step electron-transfer processes that involve the adsorption on to the electrodes of various reaction intermediates [241]. Diamond’s chemical inertness means that these intermediates are reluctant to adsorb; consequently, the catalytic effect for HER and OER on bare BDD electrodes is weak [240].

However, the situation changes when the BDD electrodes are decorated with nanoparticles, as these now act as the catalyst, lowering the overpotential needed for EWS, while the BDD electrodes serve as robust, corrosion-resistant, fouling-resistant but electrically active supports. For example, BDD electrodes treated with CuO and ZnO nanoparticles have been reported to increase the efficiency of the EWS process [242], while Ir-black powder on BDD electrodes has been shown to be particularly suitable for the OER [243].

An alternative approach is to use the energy from sunlight to photocatalytically split water. For example, Ashcheulov *et al*. [244] reported that Si photoelectrodes were able to convert sunlight into electrical current; however, the Si gradually degraded when in contact with the electrolyte. To prevent this, the photocurrent was passed into the aqueous electrolyte via a protective BDD coating decorated with a cobalt phosphate catalyst, and this current was sufficient to drive EWS. Similarly, nanostructured BDD electrodes covered by an *n*-type TiO_2_ thin film have been shown to develop a high photocurrent suitable for EWS [245]. Cu_2_O nanoparticles on BDD electrodes also act as efficient photocathodes for solar hydrogen generation [246]. There are a large number of possible metal and metal-oxide catalysts that can be used alongside BDD electrodes for solar-energy-driven photoelectrochemical water splitting [247], most of which are yet to be studied—so this is definitely an area to watch over the coming years.

#### (vi) Water purification

As well as detecting possible toxic compounds and ions in water sources, BDD electrodes can be used to clean up the water, removing organic toxins (chemical agents [248] or bacteria/viruses [249]) by electrolytic destruction [250]. This is an electrochemical technique that involves passing a high current (often many tens of amps) through the contaminated water via a series of electrodes. The current breaks apart any dissolved or suspended molecules, converting the organic components into CO_2_ and rendering the toxins harmless and the water safe. Unfortunately, the high currents sustained for long periods of time cause degradation and erosion of most electrodes, which must be replaced periodically at a high cost in money and time. BDD electrodes have proven to be more robust than most alternative materials, allowing higher currents to be used for longer periods between replacements [251].

One of the most environmentally important and timely water-purification challenges being tackled by BDD electrodes is to oxidatively destroy endocrine-disrupting chemicals (EDCs) in wastewater effluents [252]. EDCs are compounds that interfere with the function of the endocrine system that synthesises various regulatory hormones in mammals, fish and birds. The most common EDCs are used as pesticides (chlorpyrifos and dichlorodiphenyltrichloroethane), plastics (bisphenol A), plasticisers, industrial chemicals and solvents (dioxins, polybrominated biphenyls and polychlorinated biphenyls), surfactants (per- and polyfluoroalkyl substances) and preservatives (parabens) [253]. BDD anodes in water samples have been shown to be effective in the destruction of many of these EDCs with very high removal efficiencies, although scale-up is still an ongoing major problem [252].

A number of companies currently offer commercial water-purification systems based on BDD electrode technology, e.g. Proaqua (Austria), CSEM (Switzerland) and WaterDiam (France). The technology has recently been miniaturised, by a company called Enozo [254], to produce micro-ozone cells, which have been commercialized into household cleaning sprays, replacing bleach, alcohol or other cleaning fluids. At present, the available water-purification systems are suitable for household supplies, or perhaps a small factory, but not yet for large-scale use, e.g. to clean up rivers or reservoirs. However, in principle, to do so, this technology just needs to be scaled up and made portable.

#### (vii) Solvated electrons

If diamond is illuminated with ultraviolet light at photon energies greater than the diamond band gap of 5.5 eV (*λ* < 225 nm), electrons are excited from the valence band into the conduction band, from where they may be ejected from the surface. For H-terminated diamond, the surface dipole formed by the electronegativity difference between H and C, gives rise to negative electron affinity (NEA). This NEA surface means that no potential barrier needs to be overcome for these conduction-band electrons to escape the surface. If a liquid (rather than air or a vacuum) is present above the surface, the high-energy ejected electrons can interact with the liquid molecules to form ‘solvated electrons’—complexes where isolated electrons are surrounded and stabilized by polar solvent molecules [255]. These solvated electrons are extremely reactive reducing agents (−4.6 V compared to a normal hydrogen electrode) that can be used to drive chemical and biological reactions that are not accessible through conventional methods.

In 2013, Hamers’ group at the University of Madison–Wisconsin reported the use of transient absorption spectroscopy to detect solvated electrons arising from emission of electrons from diamond into water [256]. The diamond sample was irradiated with a pulsed 213 nm UV laser while the change in intensity of a second laser beam (633 nm) parallel to the diamond surface was detected. A decrease in intensity of the transmitted 633 nm light was seen as a result of absorption by the newly created solvated electrons.

Solvated electrons generated by diamond surfaces or nanoparticles have been reported to expedite a number of reactions [257]. An example is the reaction of N_2_ gas and H_2_ gas to form NH_3_, which is normally very demanding and expensive due to the high-energy intermediates involved and the reluctance of the two reactants to adsorb on to a solid catalyst or electrode. Using solvated electrons by-passes these problems [258]. NH_3_ production has also been demonstrated using this approach in both single-compartment and dual-compartment cells [259]. This photo-electrocatalysis reaction has been further improved by using NH_2_-terminated diamond [260] and also by embedding 100 nm Ag nanoparticles into the BDD electrodes [261]. Reduction of CO_2_ to CO was also demonstrated with 95% efficiency using solvated electrons generated from diamond electrodes [262]. However, to sound a note of caution—after the initial excitement about the possibilities of solvated electron chemistry in the early 2010s, in the decade following, surprisingly few reports of new reactions using solvated electrons from diamond electrons have appeared, although a few intriguing publications still appear occasionally [263].

### (b) Electronic devices

Diamond has a number of advantages when compared to other wide-band-gap semiconductors, such as SiC or GaN, due to its high hole and electron mobilities (greater than 2000 cm^2^ V^−1^ s^−1^), high critical electric field (greater than 10 MV cm^−1^), extremely high thermal conductivity (approx. 22 W cm^−1^ K^−1^) and very wide band gap (5.4 eV) [264]. As a result, there is a large variety of applications in the medium-to-high-power regime for which diamond would be the ideal semiconducting material (see the excellent reviews by Donato *et al*. [265] and Araujo *et al*. [266] and the comprehensive book by Koizumi *et al*. [267]). At the moment, many of these applications might be considered niche and are limited to high-power, high-frequency and/or high-temperature operations. Nevertheless, improved efficiency in power electronics is becoming increasingly important as countries become more environmentally aware. This is because processing electricity along the chain from primary sources (power stations, solar farms, etc.) to end-users (factories, houses) loses approximately 9% of the generated energy [268], the majority of which comes from the use of inefficient Si-based power-conversion systems. Replacing Si power converters with diamond ones, which can switch higher power loads with fewer losses and at higher frequencies, could reduce the energy wastage significantly.

There are several problems that have held back progress in diamond electronics for over 30 years. The most important and frustrating of these is the continued lack of an effective room-temperature *n*-type conductor (see §8), which is crucial for fabrication of many important types of devices. Progress has also been hindered by the painfully slow progress in commercializing large-area SCD wafers. In comparison, the standard size SiC wafer produced today is 150 mm (6 inches), and many fabrication facilities are already moving to 200 mm (8 inches) wafers. Similarly, GaN-on-Si wafers can be purchased relatively inexpensively at all sizes up to 200 mm, and even the relative newcomer, gallium oxide, can be purchased at sizes up to 100 mm (4 inches). Although in recent years a lot of progress has been made with increasing the size of diamond wafers (see §11), the SCD substrates that are commercially available now are approximately 1 inch (25 mm), which is significantly smaller than competing materials, and are considerably more expensive. Sizes will increase and prices will fall, but the question remains whether the semiconductor industry will wait for diamond to catch up, or ‘make do’ with the existing materials because they are available now and ‘good enough’ for most applications.

#### (i) Schottky diodes

Attendees at any international conference about diamond science and technology will find that there will be several sessions devoted to devices, and maybe half of the presentations will describe diamond Schottky diode (SD) devices [269]. The reason for this focus on SDs results from the lack of a suitable *n*-type dopant for diamond—SDs only require *p*-type diamond and so are the obvious candidate to demonstrate workable, commercial diamond devices.

An SD is a semiconductor diode formed by the junction of a semiconductor with a metal. As a diode, it conducts current primarily in only one direction where it has low electrical resistance and therefore a low forward voltage needed to pass current. In the reverse direction, there is a high resistance that prevents backward current flow. If the semiconductor is *p*-type diamond, then the forward voltage can be very low, while the reverse voltage is extremely high, allowing efficient switching of high-power systems operating at up to 20 A (equivalent to a current density of 100 A cm^−2^) at 500 K [270].

**Figure 17.**
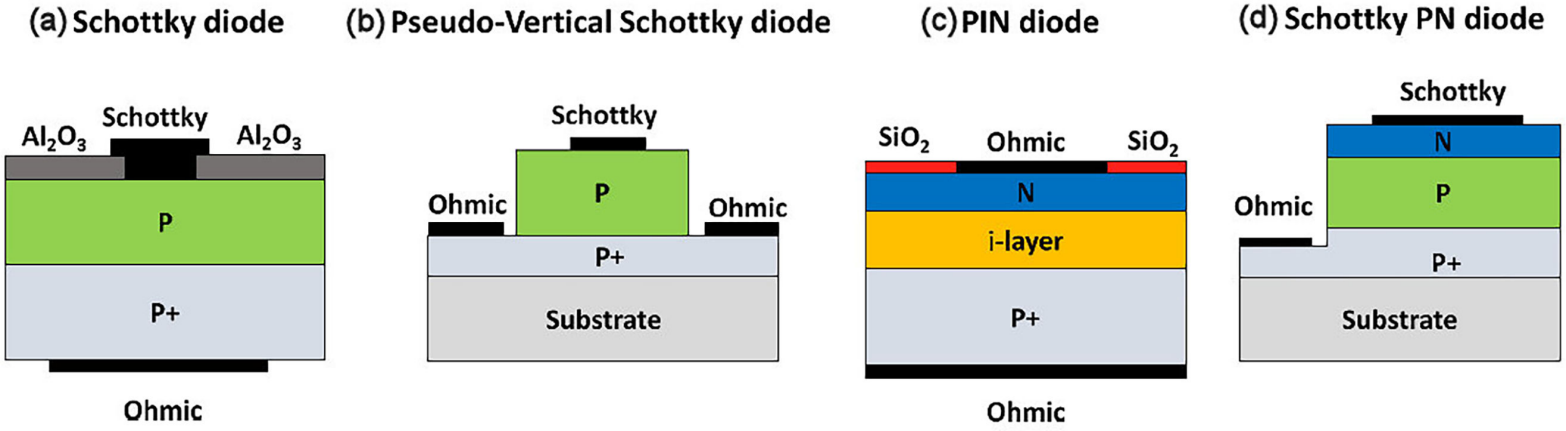
Different vertical device structures for diamond diodes. (a) Vertical SD using only *p*-type diamond. (b) Pseudo-vertical SD that uses only *p*-type diamond but no back contact. (c) PIN diode composed of a sandwich of *p^+^*-type diamond, an undoped intrinsic diamond layer and an *n*-type diamond layer, with Ohmic contacts on either side. (d) Schottky PN diode. Figure reproduced from [265] and modified under the Creative Commons Attribution 3.0 licence.

The vertical structure (as shown in figure 17) is one of the best geometrical configurations used for diamond SDs due to its low serial resistance. This structure is characterised by vertical electrical current transport through a layered stack comprising an active layer and a highly conductive substrate.

The problems with obtaining large-area SCD have resulted in the development of pseudo-vertical diamond SDs (figure 17b), where heavily doped p^++^ diamond layers are grown on top of a diamond substrate. A range of metals (W, Zr, Cu and Pt) and surface treatments have been studied to improve the uniformity of the diamond-metal junction, as well as to reduce leakage currents which result in premature breakdown of the device. Using Zr metal, a pseudo-vertical diamond SD has been demonstrated operating at 1000 A cm^−2^ at 6 V and a high reverse breakdown field greater than 7.7 MV cm^−1^ [271].

Despite the difficulty with *n*-type doping, it is still possible to make diodes using *n*-type diamond; however, the high resistance of the p*–*n junction often limits the efficiency of these types of devices [272]. Nevertheless, various so-called PIN devices and Schottky PN diodes (figure 17c,d) have been fabricated with reasonable performance figures [265].

#### (ii) Field effect transistors

Field effect transistors (FETs) are devices that control the flow of current from one electrode (the source) to another (the drain) by the application of a voltage to a third electrode (the gate). The applied voltage alters the conductivity between the drain and source, allowing the FET to be used as a digital on/off switch for binary electronics or as a current amplifier. FETs are unipolar transistors because they only involve single carriers, i.e. either electrons or holes, but not both. This means FETs can be fabricated using only *p*-type diamond, and the lack of an *n*-type dopant is not so critical. There are many different types of FET devices, but the three which have been applied most to diamond are surface-channel devices, δ-doped devices and two-dimensional hole-gas devices.

#### Surface-channel FET devices

**Figure 18.**
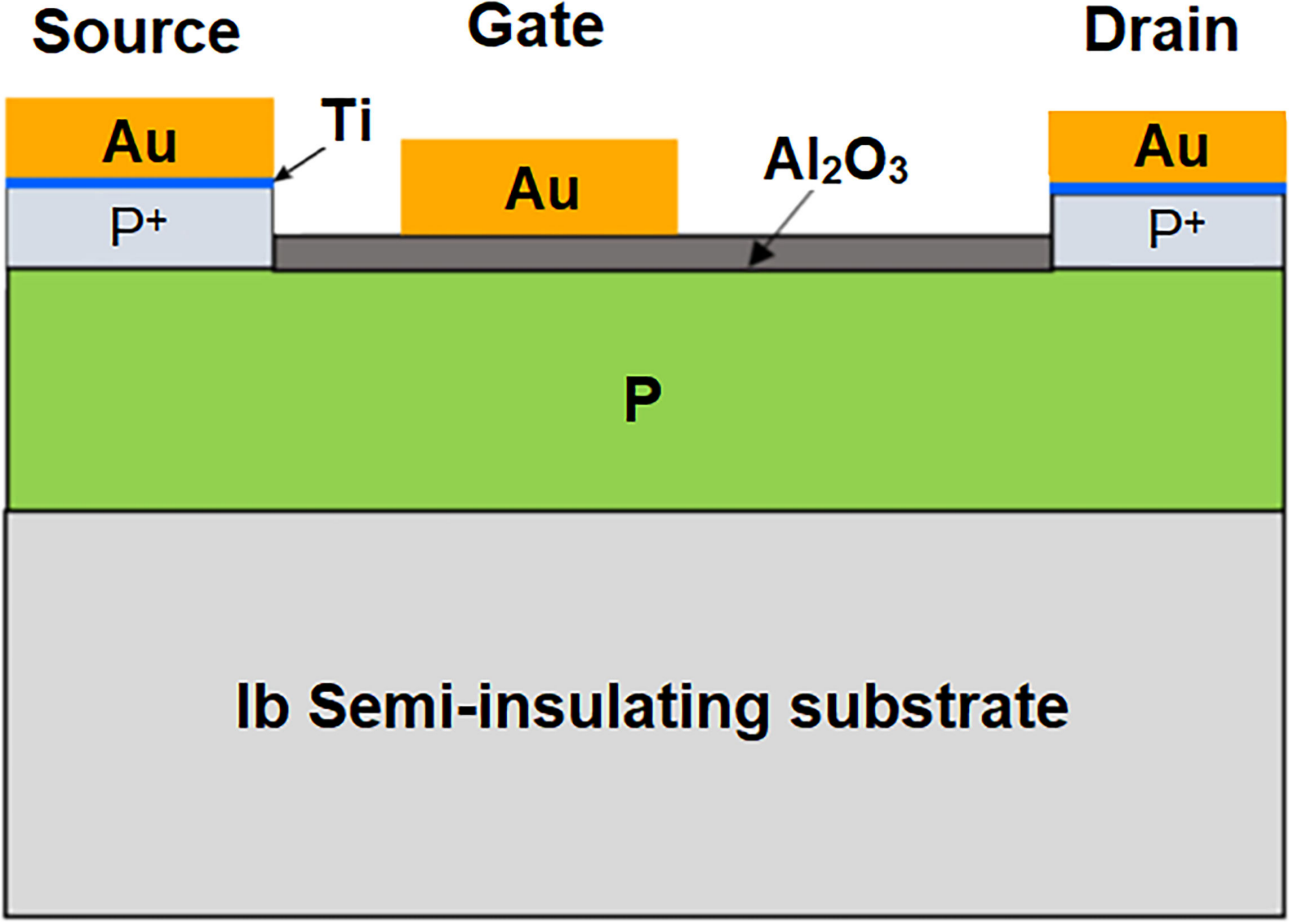
A typical design for a diamond lateral deep-depletion MOSFET-style device. The type-1b diamond substrate (usually HPHT) has a *p*-type BDD layer (green, labelled 'P') deposited homoepitaxially on to it. The source and drain electrodes are composed of heavily BDD (grey, labelled P^+^) capped with a highly conducting metal such as Au (gold), with a thin Ti layer (blue) at the interface allowing a good electrical contact to be formed with the underlying diamond. These are patterned using standard photolithography and dry etching methods. The Au gate electrode is electrically isolated from the BDD layer by a gate oxide (grey), in this case made from insulating Al_2_O_3_. Image reproduced and modified from [265]. Creative Commons Attribution 3.0 licence.

The most common type of FET device uses the metal-oxide-semiconductor (MOS) structure for the gate electrode, where a metallically conducting gate electrode sits upon a very thin oxide layer on top of a semiconductor (in this case, doped diamond) substrate (figure 18). Thus, the gate has a capacitor structure with the oxide preventing direct electrical contact between the metal and semiconductor. When an appropriate voltage is applied to the gate, current flows from the source to the drain through a thin conducting channel that has been temporarily created close to the diamond surface beneath the gate oxide.

A number of MOSFET devices have been fabricated using diamond as the semiconductor, with varying degrees of success. However, the lack of a solid oxide for diamond often results in highly defective interfaces, which negatively affects the carrier mobility through the source-drain channel. Numerous diamond–metal-oxide interfaces have been studied over the past few years [273], with Al_2_O_3_ exhibiting the best performance [274]. The difficulty in making standard MOS devices has led to new designs such as finFETs operating in so-called deep-depletion mode [275], with reasonable device performance but a rather high negative threshold voltage, suggesting that a high level of interface defects remained.

The gate-oxide problem can be eliminated by using different types of device designs that do not require a gate-oxide layer, such as junction FETs, metal-semiconductor FETs and bipolar transistors. The performance of all these devices is reviewed in detail in [265], and improvements are continually being made at a slow and steady pace. However, competing materials, in particular SiC, have more than a decade’s head start and may well capture the market for high-power, high-temperature devices *before* diamond devices become commercially viable.

#### δ-Doped devices

So-called ‘delta doping’ requires the epitaxial growth of a very thin (less than 2 nm), heavily boron-doped diamond layer with B concentration above the metallic transition point (see figure 10 earlier). This doped layer is called a ‘delta-doped layer’ from its similarity to the abrupt ‘δ-function’ in mathematics because it has atomically sharp and atomically smooth interfaces between itself and the undoped diamond layers above and below it. Although a number of research groups have attempted to make δ-doped FET devices [267,276], attempts at achieving a true monolayer doping profile with significant mobility enhancement have generally been disappointing, mainly due to the difficulty of controlling the dopant concentration profile to such precision.

#### Two-dimensional hole-gas field effect transistor devices

**Figure 19.**
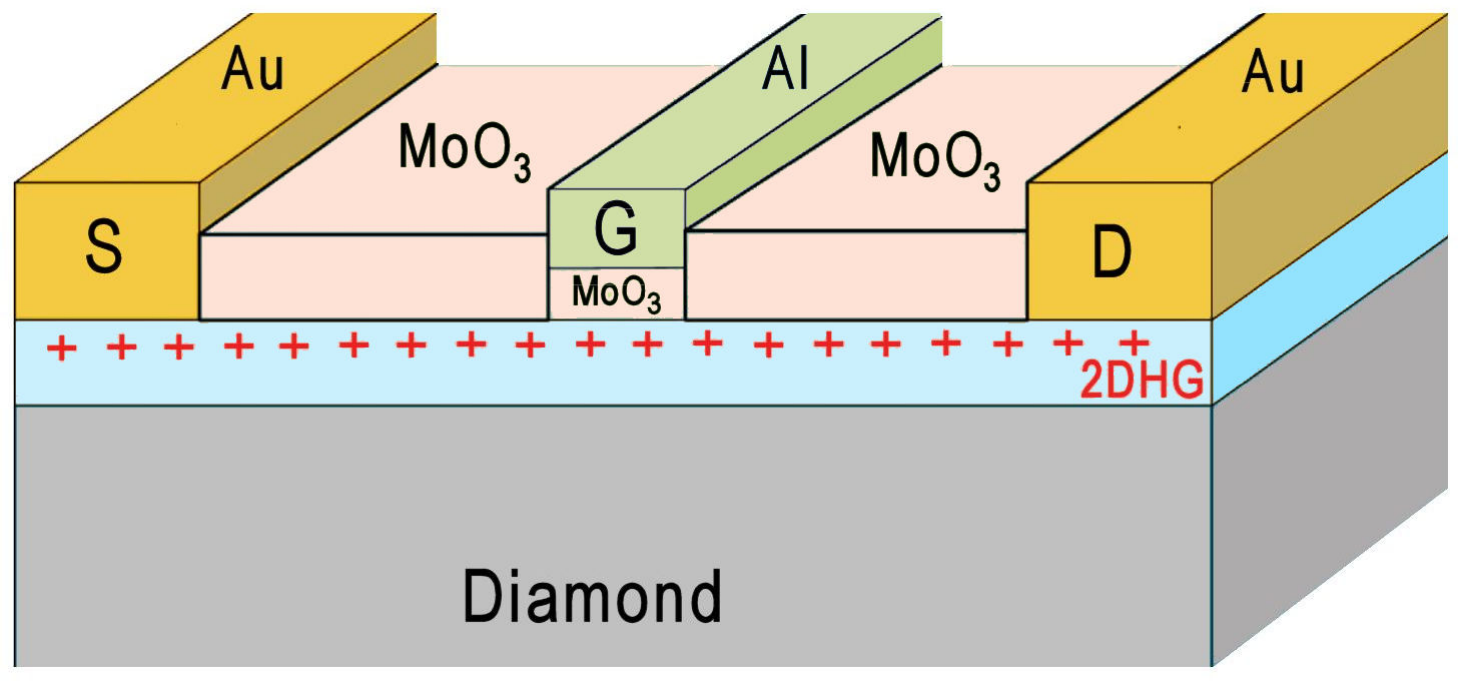
Schematic cross-sectional view of a diamond-based MOSFET device utilizing the 2DHG conduction channel in H-terminated diamond protected by a MoO_3_ capping layer, based on the design proposed in [156]. S, G and D refer to source, gate and drain, respectively.

Research groups, such as those at the University of Glasgow [154] and Waseda University (Tokyo) [277], have been exploiting surface transfer doping in hydrogenated diamond (described earlier in §8), to produce novel FET devices in which the conduction from source to drain, moderated via a gate electrode, is through the 2DHG conductive layer, rather than through the semiconducting substrate (figure 19). Not only do these 2DHG FETs exhibit excellent device performance, but because they are fabricated directly on to an insulating diamond substrate, they should be considerably more radiation hard compared to standard Si-based devices.

For all the SD and FET devices mentioned above, the comprehensive analysis conducted by Donato *et al*. [265] has suggested that diamond may remain a niche device material for quite some time and be limited to devices with high junction temperatures (greater than 450 K), medium-high frequency (greater than 20 kHz) and high voltage (greater than 3 kV) where they should outperform SiC and GaN commercial alternatives. More specialized diamond electronic devices, such as radiation-hard architectures, surface acoustic wave filters and micro/nanoelectro-mechanical systems, are discussed in the electronic supplementary material.

### (c) Quantum applications

Diamond-based quantum technologies revolve around optically active atomic-scale defects in the diamond lattice [278]. The most studied is the NV defect (see §9) [279], although various other defects with similar properties have also been investigated [280]. The electrons associated with these defects have a spin that is sensitive to external magnetic fields, and due to diamond’s transparency, the electrons can be manipulated using light. Moreover, these spins possess long coherence times (i.e. the duration for which the spin remains stable and predictable) due to their exceptional isolation from other nearby NV-centre spins by the diamond lattice. This isolation has another important consequence; because the NV centre acts like a single isolated pseudo-atom, it can only absorb or re-emit *one* photon at a time. So, when a laser beam is used to probe the defect, only *one* photon from the billions of laser photons is absorbed, and its energy is used to excite *one* electron. After a short delay, the electron relaxes to a lower state, emitting *only one* photon (usually of lower energy than the original laser photons). In the case of the NV centre, usually, the pumping photons are green (*λ* = 532 nm), while the emitted fluorescence is red (*λ* = 637 nm) [281].

The process by which the spin is measured is called optically detected magnetic resonance. The NV defects have a remarkable set of energy levels, in that no matter what ground-state spin an electron has originally, if the diamond is illuminated with green light the electron will cycle through all the allowed energy levels, absorbing green photons and then relaxing and re-emitting red photons. Due to quantum selection rules and the relative lifetimes of the states involved, eventually, the electron is statistically more likely to end up in the ground state with spin *m*_s_ = 0. For multiple NV centres, if the electrons are cycled around this loop enough times, nearly all their electrons will end up in the same *m*_s_ = 0 state, and the spins of each NV centre will be aligned. This effectively ‘resets’ the system, ensuring the starting point is always identical.

Once reset like this, the readout from a quantum experiment is observed as a change in the red fluorescence after shining green light on a single NV defect, or on to a group of them, while scanning an applied MW field. When the MW frequency hits resonance with an *m*_s_ = 0 to *m*_s_ = ± 1 transition, a decrease in fluorescence intensity is observed. So, by measuring the intensity of the fluorescence, the spin state of the defect can be read out.

The quantum-mechanical nature of spin means that the red fluorescence photon will be emitted with spin properties based on those of the original electron, for example, either ‘up’ or ‘down’. The single photons emitted from different NV centres can be captured in fibre-optic waveguides and then combined to create quantum superpositions and entanglement. Such entangled photons form quantum bits (qubits) that are the key to quantum communication and computing.

#### (i) Quantum computing

**Figure 20.**
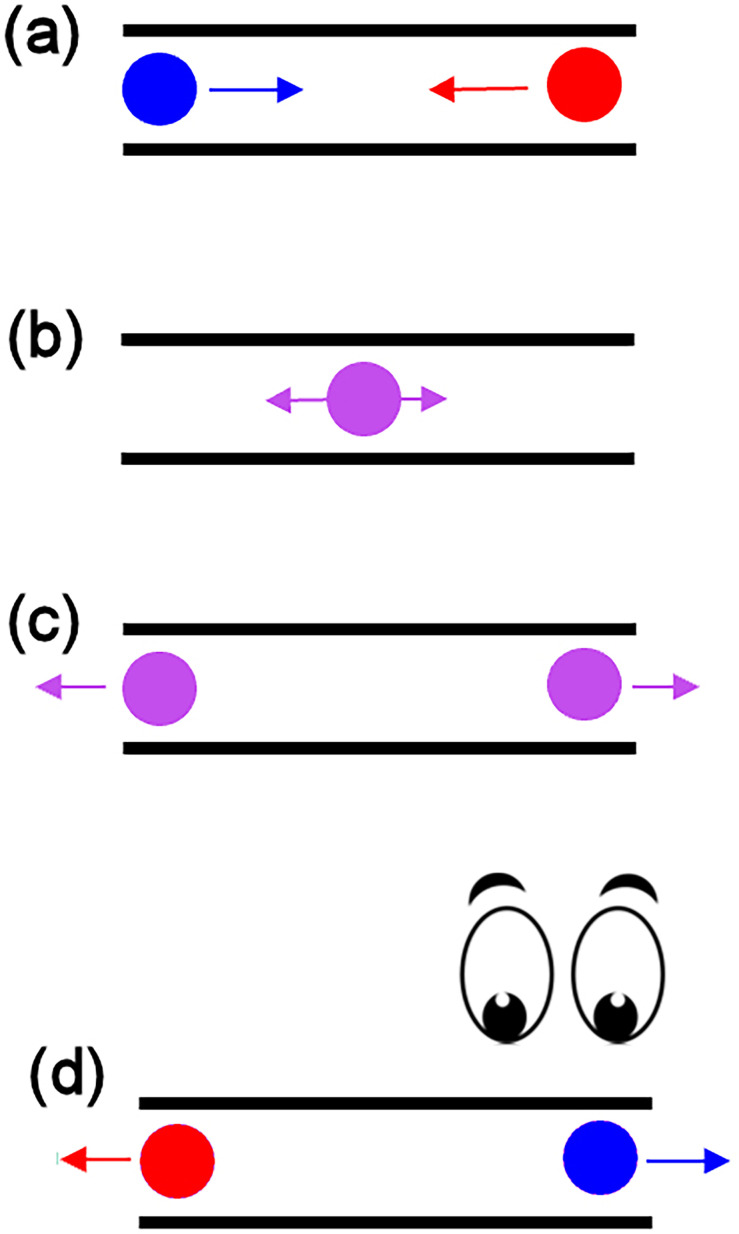
A simplistic representation of quantum entanglement of two photons. (a) Two independent photons with opposite spin (depicted as red and blue) emitted from different NV centres approach each other inside a fibre-optic waveguide. (b) When they meet, their wavefunctions entangle, and they both share the same superposition wavefunction*—*i.e. the photons are now both red *and* blue simultaneously (depicted as purple). (c) When they separate, both photons remain entangled in the same shared quantum state. (d) If one of the photons is observed, the superposition wavefunction collapses, and the spin of that photon is fixed at that instant as being either up or down (red or blue, in this case red). At that same instant, the spin of the other photon is also known (blue), even though it was never observed and might now be thousands of kilometres from its partner.

In standard binary computing, a ‘bit’ is either 1 or 0. If spin-up is a 1 and spin-down is a 0, then two entangled photons in a qubit would be both 1 *and* 0 at the same time, until one of the pair is examined (figure 20). At the instant the spin of the examined photon is revealed, the spin of the other photon is also known, even though that second photon had never been examined and may now be a very long distance from the first photon. (In fact, quantum theory states that there is *no* limit to this separation—the two photons might be on different continents, different planets or even different galaxies—yet the communication between them remains instantaneous.)

The power of quantum computing lies in the fact that changing one of the states of *one* of the qubits immediately changes the states of *all* of its entangled partners. This means that the number of calculations that can be performed scales as 2^*n*^, where *n* is the number of qubits. So, entangling only 20 qubits together enables the equivalent of over a million calculations to be performed instantly. This means that a calculation performed linearly by a normal computer, which requires multiple steps (additions, multiplications, carrying values, etc.), can be done in parallel in *one* fast step by a quantum computer. Thus, quantum computers have the potential for unprecedented computing speeds and, coupled with artificial intelligence, may truly be world-changing.

At the time of writing, the World’s most powerful quantum computer is reported to be the *Google* Sycamore processor with 70 (non-diamond) qubits. In 2019, an earlier version of Sycamore with only 53 qubits completed a calculation in 200 seconds that *Google* claimed [282] would take a normal state-of-the-art supercomputer 10,000 years to finish. Although astonishing, current quantum computers do not yet use diamond defects to create qubits and so need to be cooled by liquid helium to temperatures a few degrees above absolute zero to attain coherence times sufficient for complex calculations. Diamond-based quantum computers should work at *room temperature*, making them vastly cheaper and easier to operate.

Progress in the field of diamond quantum computing has been fairly rapid since the first demonstration of entanglement between an NV spin and a photon was reported in 2010 [283]. In 2013, entanglement was reported between two separated NV centres using lenses patterned into the diamond surface acting as amplifiers [284]. Later that year, the distance between the qubit photons (i.e. the ‘node’ separation) was extended to 3 m [285]. In 2015, the node separation was extended to more than 1 km [286]. In 2019, a group at Delft University reported a 10-qubit register that could store quantum information for up to 75 s [287]. Such long timescales provide sufficient time for controlled logical operations (AND, NOR, etc.) to be performed between different spin qubits [288]. The initialization and manipulation of the diamond NV centre spins can now be performed with single-shot measurements, making them ideal systems for scaling up into multi-qubit quantum registers [289].

Research in this area is now following two approaches. The first aims to further improve the quality of the diamond, reducing defects and developing methods to control the density and positioning of NV (or other) centres, along with maximizing the efficiency by which photons are extracted from individual NVs [290]. The second approach involves developing the new software required to control these quantum systems. In particular, code that enables errors in qubit states to be detected and corrected has already been realized in small qubit networks [291]. The key challenge for the future is to scale up to quantum networks of tens or hundreds of qubits. Such work is now proceeding rapidly; however, it may be many more years before we see a fully functioning diamond quantum computer [292].

#### (ii) Quantum cryptography

Another application of the control and entanglement of photon spins from the NV centre is to enable perfectly secure communications [293]. To do so, the information being sent would be encrypted using an entangled quantum key known only to the sender and receiver. If the message were to be intercepted by a third party, the wavefunction of the quantum key would collapse and the attempted eavesdropping event revealed. Such secure communications should, in theory, make copying or modifying confidential data (e.g. a PIN number sent over the internet or medical records) almost impossible [278].

#### (iii) Quantum sensing and magnetometry

Electronic spins in NV centres are sensitive to temperature, electric fields and magnetic fields and can be used as highly sensitive sensors that operate at room temperature. Most of the work in this area concerns magnetometry and describes the measurement of tiny magnetic fields at the nanometre scale, for example, in the study of new superconductors or semiconductor materials.

Quantum sensing is achieved in practice by placing or depositing on to the substrate a diamond sample with a high-density, thin layer of near-surface NV centres. The NV centres can also be incorporated into nanostructures, such as DND particles, to achieve nanometre proximity to the site of interest, or attached to fibres to make magnetic endoscopes. For instance, DND particles containing NV centres have been ingested by biological cells enabling *in situ* sensing and tracking [162], while the local temperature inside a living cell has been monitored providing information about the cell’s metabolism and transport pathways [294]. Indeed, since the first reports of NV-based magnetic sensing in 2008 [295–297], this technique has been used to detect a range of other physical quantities, such as electric fields, charge, voltage, current, orientation, strain, temperature and pressure. A summary of different sensor types with their related sensitivities is listed in table 2 of Nebel’s detailed review paper [279].

Looking to the future, there is a growing number of companies developing diamond quantum technology, including large companies such as Lockheed Martin, Bosch and Thales, as well as many start-ups such as Quantum Diamond Technologies [298], NVision [299] and Qnami [300]. In parallel with the development of high-purity substrates within which NV centres (or other suitable defects) can be fabricated, quantum technology could well become one of the killer applications for diamond technology in the second half of this century.

## 13. Conclusions

The advances in CVD diamond technology which have occurred over the past decade have mostly been driven by the upsurge in the market for lab-grown diamond jewellery, which has seen huge increases in the number of SCD gemstones produced, their size, quality and throughput. Countries including, Israel, the US and especially China, and more recently, India, have invested heavily in the technology and equipment necessary for mass-market production of lab-grown diamonds. The market for CVD lab-grown diamond jewellery is still increasing at a rapid rate and may exceed 20% of all global diamond jewellery sales by the end of the decade. Lab-grown HPHT and CVD diamonds are already available in a range of colours [197] and at sizes previously only affordable by the very wealthy.

However, the next few years will probably witness the maturing of the CVD diamond gem industry, driven by (i) nearly limitless supply, (ii) uniform and reproducible high quality, (iii) branding and proprietary design and (iv) even more unusual custom shapes and colours [301]. With the market for smaller gemstone diamonds eventually becoming saturated, manufacturers may switch tactics from quantity to size. It has been estimated [301] that by the end of the decade, a customer with a typical $6500 budget may be able to purchase a 6- or even 7-carat lab-grown diamond with a diameter greater than 1.2 cm.

An alternative strategy to ‘bigger is better’ may be to use lab-grown diamond gems in a way that is impossible with natural diamonds. With advances in growth and laser-cutting technology, manufacturing an ‘all-diamond’ ring or bracelet may become possible. The conventional cuts (such as the well-known ‘brilliant’ cut) that have been used for centuries to enhance the sparkle of natural stones may find new rivalry in the form of complex 3D custom cuts or shapes that differ considerably from those traditional ones. Similarly, by treating the diamonds post-deposition using HPHT annealing or radiation exposure, diamonds might be fabricated in a range of exotic colours that do not exist in nature.

The developments in the gem industry so far have fed into the scientific sector in terms of ultra-pure SCD substrates with increasingly larger wafer sizes for electronic and quantum applications and at progressively lower costs. But increased supply means that prices of CVD diamond gemstones are falling year-on-year [302], although the ‘green’ credentials of lab-grown diamonds combined with the consumer’s certainty that they do not come from conflict zones or are mined by an exploited labour force should prevent the price dropping too low. If that predicted trend continues, profit margins will eventually drop, possibly leading to a decrease in investment in diamond technology by the gem manufacturers. The knock-on effect for scientific diamond research may be less research funding, potentially leading to a much slower delivery time for many of the more ambitious applications mentioned in §12. It is somewhat ironic that a material with so many superlative properties and exciting potential scientific applications may have its future determined by the whims of fashion!

Nevertheless, there is a renewed hope that the three ‘killer applications’, electrochemistry, quantum information and high-temperature electronics, along with the huge number of possible diverse applications for CVD diamond that are beginning to find their way to market [5], might be sufficient for diamond research to flourish—despite the uncertainties of the gemstone market. The original review paper in 2000 was entitled: *Diamond—a 21^st^-Century Material*. Maybe that now needs to be qualified somewhat, to: *Diamond—a mid-21^st^-Century Material*, because it may take another 20 years or so before diamond can finally live up to its promise of a ubiquitous wonder material.

## Data Availability

Supplementary material is available on the University of Bristol Repository [303]. Supplementary material is also available online [304].
